# Bispecific antibodies (anti-mPEG/anti-HER2) for active tumor targeting of docetaxel (DTX)-loaded mPEGylated nanocarriers to enhance the chemotherapeutic efficacy of HER2-overexpressing tumors

**DOI:** 10.1080/10717544.2018.1466936

**Published:** 2018-05-02

**Authors:** Chia-Yu Su, Michael Chen, Ling-Chun Chen, Yuan-Soon Ho, Hsiu-O Ho, Shyr-Yi Lin, Kuo-Hsiang Chuang, Ming-Thau Sheu

**Affiliations:** a School of Pharmacy, College of Pharmacy, Taipei Medical University, Taipei, Taiwan, ROC;; b Ph.D. Program in Clinical Drug Development of Chinese Herbal Medicine, Taipei Medical University, Taipei, Taiwan, ROC;; c Department of Biotechnology and Pharmaceutical Technology, Yuanpei University of Medical Technology, Hsinchu, Taiwan, ROC;; d Graduate Institute of Medical Sciences, College of Medical Science and Technology, Taipei Medical University, Taipei, Taiwan, ROC;; e Department of Primary Care Medicine, Taipei Medical University Hospital, Taipei, Taiwan, ROC;; f Department of General Medicine, School of Medicine, College of Medicine, Taipei Medical University, Taipei, Taiwan, ROC;; g TMU Research Center of Cancer Translational Medicine, Taipei Medical University, Taipei, Taiwan, ROC;; h Graduate Institute of Pharmacognosy, Taipei Medical University, Taipei, Taiwan, ROC

**Keywords:** Bispecific antibody, docetaxel, mPEGylated nanocarriers, active targeting, chemotherapeutics

## Abstract

Anti-mPEG/anti-human epidermal growth factor receptor 2 (HER2) bispecific antibodies (BsAbs) non-covalently bound to a docetaxel (DTX)-loaded mPEGylated lecithin-stabilized micellar drug delivery system (L*_sb_*MDDs) were endowed with active targetability to improve the chemotherapeutic efficacy of DTX. DTX-loaded mPEGylated L*_sb_*MDDs formulations were prepared using lecithin/DSPE-PEG(2K or 5K) nanosuspensions to hydrate the thin film, and then they were subjected to ultrasonication. Two BsAbs (anti-mPEG/anti-DNS or anti-HER2) were simply mixed with the L*_sb_*MDDs to form BsAbs-L*_sb_*MDDs formulations, respectively, referred as the DNS-L*_sb_*MDDs and HER2-L*_sb_*MDDs. Results demonstrated that the physical characteristics of the BsAbs-L*_sb_*MDDs were similar to those of the plain L*_sb_*MDDs but more slowly released DTX than that from the L*_sb_*MDDs. Results also showed that the HER2-L*_sb_*MDDs suppressed the growth of HER2-expressing MCF-7/HER2 tumors, increasing the amount taken up *via* an endocytosis pathway leading to high drug accumulation and longer retention in the tumor. In conclusion, the BsAbs-L*_sb_*MDDs preserved the physical properties of the L*_sb_*MDDs and actively targeted tumors with a drug cargo to enhance drug accumulation in tumors leading to greater antitumor activity against antigen-positive tumors.

## 1. Introduction

In the past few decades of rapidly evolving drug research, numerous high-potency chemotherapeutic drugs have been discovered. However, notwithstanding the rapid progress in drug innovations, cancer drugs have gained a reputation for having high risks with little chance of efficacy. These are mainly attributed to the following causes: (i) many potent drugs are highly hydrophobic which keeps them from being used in the clinic; (ii) a lack of specificity of chemotherapeutic drugs also causes high toxicity to normal cells; (iii) an unsuitable biodistribution following an intravenous (IV) injection for most drugs results in low therapeutic efficacy and adverse effects; and (iv) excipients used to enhance the solubility of the drug formulations might cause additional toxicities in patients (Chabner & Roberts, 2005). For the success of chemotherapeutic agents in clinical applications, a durable and specific drug delivery system is required to carry and release the drugs into the right pathological site (Peer et al., 2007; Brigger et al., 2012). For this purpose, numerous nanocarrier (NC) types like liposomes, micelles, polymeric nanoparticles (NPs), dendrimers, solid-lipid nanoparticles (SLNs), and gold NPs have been investigated for controlled drug release applications (Davis et al., 2008; Wang et al., 2012). These NCs delivery systems can through a leaky tumor blood vasculature *via* an enhanced permeability and retention (EPR) effect. Also, further modification of stealthy decorations on the surface with polyethylene glycol (PEG) offers to reduce immunogenicity and prolong the circulation times. Those advances in nanomedicine have demonstrated obvious advantages, including preferential drug accumulation in tumor sites, decreased side effects, better drug tolerance, and improved patient compliance in clinical practice (Zhong et al., 2014; Hare et al., 2017).

Recently, robust and emerging drug delivery systems known as lipid-polymer hybrid nanoparticles (LPHNs) which take advantage of the unique strengths of liposomes and polymeric NPs have successfully application in anti-cancer drug, anti-microbial agent, and nucleic acid (Krishnamurthy et al., 2015; Dave et al., 2017). However, LPHNs still have two problems of low encapsulation efficiency (EE) and drug loading (DL) needed to be overcome (Li et al., 2017). In our previous study, we have established high EE and drug-loaded lecithin-stabilized micellar drug delivery system (L*_sb_*MDDs) that has a polymeric core and a lipid shell to delivery hydrophobic drug to enhance antitumor efficacy and reduce systemic side effect (Su et al., 2018).

For additionally improving drug accumulation and high tumor cell specificity, the surface of the NCs can be modified by specific tumor-targeted ligands such as monoclonal antibodies (Abs; mAbs), aptamers, peptides, small molecules, and so on, to achieve active tumor targeting (Allen, 2002). Ligand-directed active targeting of NP drug formulations present improved therapeutic performances compared to their passive targeting counterparts in preclinical study (Koo et al., 2011; Nicolas et al., 2013). Among actively targeted ligands, various forms of Abs such as mAbs, antigen-binding fragment (Fab), and single-chain variable fragment (scFv)) are frequently used as efficient targeting moieties due to its nanomolar affinity and high specificity to tumor antigens (Kamaly et al., 2012; Tietze et al., 2017). A number of NCs conjugated with Abs have been developed to target tumor which highly expressing a particular surface marker, such as human epidermal growth factor receptor 2 (HER2), epidermal growth factor receptor (EGFR), and vascular endothelial growth factor (VEGF). Nonetheless, chemical conjugation of Abs to NCs might have the disadvantages of heterogeneous coupling orientations, antigen-binding function damage, altered physical properties of NCs can be altered, and it is laborious and time-consuming (Manjappa et al., 2011; Zhong et al., 2014). To avoid the problems of chemical conjugation, Kao et al. offered a simple one-step method to confer tumor specificity to methoxyl PEG (mPEG)-NCs by non-covalently bound with anti-mPEG/anti-tumor bispecific antibodies (BsAbs). BsAbs-PEG-NC showed increased drug accumulation and enhanced therapeutic efficacy in EGFR^+^ colon tumor-bearing mice (Kao et al., 2014). Thus, the mPEGylated NCs modification with anti-mPEG/anti-tumor BsAb can acquire specific tumor-targeting without further chemical coupling reactions.

**Figure 1.**
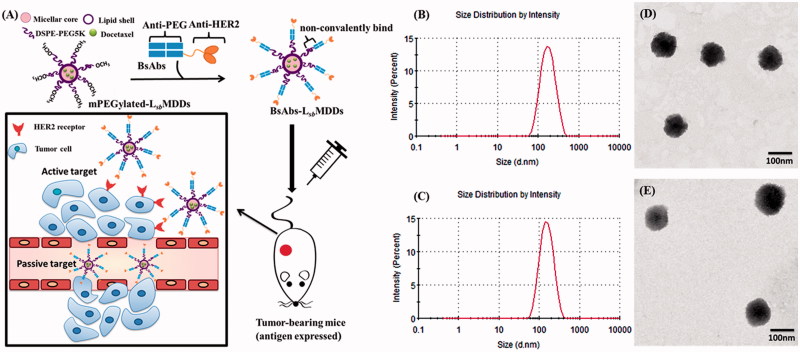
(A) Strategy of bispecific antibodies (BsAbs and anti-mPEG/anti-HER2) non-covalently bound to a mPEGylated L*_sb_*MDDs to form the BsAbs-L*_sb_*MDDs, which can specifically target antigen-expressing cancer cells by passive and active targeting. (B) Size distributions of L*_sb_*MDDs(2K) and (C) L*_sb_*MDDs(5K) were measured using dynamic light scattering (DLS) and TEM micrographs of (D) the L*_sb_*MDDs(2K) and (E) L*_sb_*MDDs(5K) (scale bars =100 nm).

Herein we established active HER2 targeting mPEGylated lecithin-stabilized micellar drug delivery system (HER2-L*_sb_*MDDs) loading with docetaxel (DTX), a first line chemotherapeutic agent for breast cancer (BC). Humanized anti-mPEG/anti-HER2 BsAbs non-covalently bound to DTX-loaded L*_sb_*MDDs to enhance tumor accumulation and improve the chemotherapeutic efficacy against HER2-positive BC is schematically shown in Figure 1(A). The L*_sb_*MDDs was incorporated with 1,2-Distearoyl-*sn*-glycero-3-phosphoethanolamine-N-[methoxy(polyethylene glycol) (DSPE-PEG) with different molecular weights (2K or 5K) by ultrasonication in a lecithin nanosuspension to fuse the supported lipid layers onto the micellar core. The anti-mPEG/anti-HER2 BsAbs consisted of a Fab fragment to attach to the methoxy ends of the mPEG on L*_sb_*MDDs surface, and a scFv targeting the HER2 on tumor cells. In this study, uptake mechanisms of the anti-mPEG/anti-HER2 BsAb-unbound/bound DTX-loaded L*_sb_*MDDs were examined, and their physical characteristics were evaluated, including the particle size and distribution, morphology, optimal BsAbs/mPEG molar ratio, cell viability, *in vitro* drug release, and biopharmaceutical characteristics of the tumor proliferation inhibition, pharmacokinetics (PK), and biodistribution.

## 2. Materials and methods

### 2.1. Materials

DTX was supplied by Qilu Pharmaceutical (Jinan, China). DSPE-PEG2K was purchased from NOF (Tokyo, Japan). DSPE-PEG5K was obtained from Avanti Polar Lipids. (Alabaster, AL). 3,3′-Dioctadecyloxacarbocyanine perchlorate (DIO), nystatin, and sucrose were obtained from Sigma-Aldrich (St. Louis, MO). 1,1’-Dioctadecyltetramethyl indotricarbocyanine iodide) (DIR) was purchased from Perkin-Elmer (Waltham, MA). Soybean lecithin (Lipoid S-100) was obtained from Lipoid GmbH (Ludwigshafen, Germany). Chlorpromazine and cytochalasin D were supplied by Cayman Chemicals (Ann Arbor, MI). Dynasore was obtained from MedChem Express (Monmouth Junction, NJ). LysoTracker Red DND-99 and Hoechst3342 were obtained from Thermo Fisher Scientific (Waltham, MA). The anti-PEG backbone mAb AGP4 was provided by Dr. Steve R. Roffler (Academia Sinica, Taipei, Taiwan). Tynen^®^ (solvent-based DTX) is a generic product of DTX manufactured by TYY Pharmaceutical (lot no: STW1407, Taipei, Taiwan). All reagents were of analytical grade and solvents used in the high-performance liquid chromatography (HPLC) or ultra-performance liquid chromatography (UPLC)/mass spectrometry (MS/MS) analysis was of HPLC or MS grade.

### 2.2. Animals and cell lines

A BC cell line (MCF-7) that poorly expresses HER2 (HER2^-^) and HER2-overexpressing cell lines (HER2^+^), were cultured in Dulbecco’s modified Eagle’s medium Ham’s F12 (DMEM/F12) containing 10% fetal bovine serum (FBS) and 1% antibiotics (penicillin/streptomycin). Nu/nu mice (females, 5 ∼ 7 weeks old) and Sprague-Dawley (SD) rats (males, 8 ∼ 10 weeks old) were used for the animal studies. The nu/nu mice were purchased from the National Laboratory Animal Center (Taipei, Taiwan), and SD rats were purchased from BioLASCO Taiwan (Taipei, Taiwan). All animal experiments were carried out in accordance with a protocol approved by the Laboratory Animal Center of Taipei Medical University (approval no: LAC-2014-0253), and all experiments were performed in accordance with animal care guidelines.

### 2.3. Preparation of the mPEGylated L*_sb_*MDDs

The DTX-loaded L*_sb_*MDDs was prepared following previously reported procedures with minor modifications to introduce mPEGylation with DSPE-PEG2K or DSPE-PEG5K into the outer shell portion of micelles (Chen et al., 2015; Su et al., 2018). In brief, a fixed drug (DTX)/amphiphilic polymer (DSPE-PEG2K) ratio of 1:5 with the addition of an appropriate amount of TPGS as an antioxidant was first dissolved in methanol, and a thin film was formed after evaporation (Rotavapor R124; Buchi, Flawil, Switzerland) of the organic solvent. Soybean lecithin (S100) at 1000 and 375 mg of DSPE-PEG5K (or DSPE-PEG2K) were suspended in 25 mL of deionized water and then subjected to ultrasonication (VCX 750, 20 kHz, Sonics and Materials, Market Harborough, United Kingdom) to form a lecithin/DSPE-PEG (2K or 5K) nanosuspension. Then 1 mL of the lecithin/DSPE-PEG (2K or 5K) nanosuspension was used to hydrate the thin film obtained above, and the reconstituted mixture was further subjected to ultrasonication at full power for at least 5 min while maintaining a constant temperature to form lecithin-stabilized NCs in the solution. Any unencapsulated drug was discarded by filtering this NC solution through a 0.22-μm membrane (Millipore, Billerica, MA). An appropriate amount of an anti-freeze agent was added to the filtrate and then freeze-dried to obtain the dry powder form of NCs. Two amphiphilic polymers of DSPE-PEG2K and DSPE-PEG5K with different PEG chain lengths were used to formulate the DTX-loaded mPEGylated L*_sb_*MDDs, respectively, designated DTX-loaded L*_sb_*MDDs(2K) and DTX-loaded L*_sb_*MDDs(5K). To prepare the DIO (green fluorescence for *in vitro* assays)-loaded or DIR (near-infrared fluorescence for *in vivo* imaging)-loaded L*_sb_*MDDs, we followed the same procedure as that for the DTX-loaded L*_sb_*MDDs except that the DTX was replaced with either DIO or DIR.

### 2.4. Physical characterization of the DTX-loaded L*_sb_*MDDs and BsAbs-L*_sb_*MDDs

Characteristics of the DTX-loaded L*_sb_*MDDs and BsAbs-L*_sb_*MDDs including the particle size, zeta potentials (ZPs), binding activity, morphology, EE, DL are described in Supplemental information.

### 2.5. Construction and expression of BsAbs and non-covalent modification of L*_sb_*MDDs with BsAbs

The anti-mPEG/anti-HER2 BsAbs were composed of a Fab of a humanized anti-mPEG (clone 15-2b) Ab and an scFv of trastuzumab (humanized anti-HER2 Ab) as previously described with minor modifications (Kao et al., 2014). The light and heavy chains of the anti-mPEG *Fab* gene were linked by the internal ribosome entry site (IRES) sequences (Chuang et al., 2010). A (Gly_4_Ser)_3_ linker, the anti-HER2 scFv, and an 6xHis tag genes were ligated after the heavy chain of the anti-mPEG Fab. To evaluate the tumor-binding specificity, negative control (anti-mPEG/anti-dansyl (DNS)) BsAbs were generated by replacing the anti-HER2 scFv with an anti-DNS scFv that binds the small chemical hapten of DNS, which is not present in cell or body (Kao et al., 2014). *BsAb* genes were then inserted into the pLNCX vector. To mass-produce the BsAbs, plasmids were transiently transfected into the Expi293 cells, according to the manufacturer’s instructions (Thermo Fisher Scientific, Waltham, MA). After 6 d, BsAbs in the supernatant were purified using HisTrap HP columns (GE Healthcare Life Sciences, Little Chalfont, United Kingdom), dialyzed against phosphate-buffered saline (PBS, pH 7.4, 10 mM), and sterilized by 0.2-μm filtration. Concentrations of BsAbs were determined by a bicinchoninic acid (BCA) assay (Thermo Fisher Scientific, Waltham, MA).

Non-covalent modification of L*_sb_*MDDs with BsAbs to form BsAbs-L*_sb_*MDDs were simply and freshly prepared by incubation of L*_sb_*MDDs with anti-mPEG/anti-HER2 BsAbs in bovine serum albumin (BSA)/PBS buffer (0.05% w/v) at RT for 1h. DNS-L*_sb_*MDDs were generated with anti-mPEG/anti-DNS BsAbs and L*_sb_*MDDs under the same procedure, and was used as a control.

### 2.6. Detection of the BsAbs (anti-mPEG/anti-HER2)non-covalently bound to the L*_sb_*MDDs(2K or 5K) by the sandwich enzyme-linked immunosorbent assay (ELISA)

The experimental protocol to assess the presence of anti-mPEG/anti-HER2 BsAbs on the L*_sb_*MDDs(2K or 5K) is illustrated in Scheme 1 (Supplemental information). First, 96-well plates were coated with 5 μg/mL of an anti-PEG backbone mAb (AGP4) in 50 μL of 0.1 M NaHCO_3_ per well at 37 °C for 2 h, and then this was blocked with a 200 μL/well of dilution buffer containing 5% w/v skim milk in PBS at 4 °C overnight. HER2-L*_sb_*MDDs were prepared at 0.01:1 of BsAbs/mPEG molar ratio. The L*_sb_*MDDs or HER2-L*_sb_*MDDs (2K or 5K) diluted with 2% skim milk to give different DSPE-PEG molar concentrations (36, 144, and 576 nM) was added to AGP4-coated wells. After incubation for 1 h, each well was washed with PBS to remove unbound NCs. Then 0.1 μg/mL of goat anti-human immunoglobulin G (IgG) F(ab’)_2_-horseradish peroxidase (HRP) (Jackson ImmunoResearch Laboratories, West Grove, PA) in 50 μL of dilution buffer was added for another 1 h to detect the BsAbs on L*_sb_*MDDs. The plates were finally washed with PBS and then 150 μL/well of ABTS substrate was added for 30 min. Color development was measured at 405 nm (Bio-Tek, Winooski, VT).

### 2.7. Optimal ratio of BsAbs to mPEG5K on the L*_sb_*MDDs for preparation of HER2-L*_sb_*MDDs

To optimize the molar ratio of the anti-mPEG/anti-HER2 BsAbs to mPEG5K on the L*_sb_*MDDs, free BsAbs unbound to HER2-L*_sb_*MDDs was measured by ELISA method illustrates in Scheme 2 (Supplemental information). First, 96-well plates were coated with 20 μg/mL of mPEG5K-NH_2_ ligand (with a methoxy end group in the PEG chain) in 50 μL of 0.1 M NaHCO_3_ per well at 37 °C for 2 h and then blocked with 200 μL/well of dilution buffer containing 5% w/v skimmed milk in the PBS at 4 °C overnight. The HER2-L*_sb_*MDDs(5K) prepared at three different BsAbs/mPEG molar ratios (of 0.002:1, 0.01:1, and 0.02:1) with a fixed amount of the L*_sb_*MDDs(5K) was diluted to same concentrations of BsAbs, and were incubated in the wells for 1 h, followed by extensive washing each well with PBS, and then goat anti-human IgG F(ab’)_2_-HRP was added, and the procedure described in Section 2.6. The addition of only the L*_sb_*MDDs(5K) or free anti-mPEG/anti-HER2 BsAbs was, respectively, used as the negative control and positive control.

### 2.8. Tumor targeting of the mPEGylated L*_sb_*MDDs with non-covalently bound BsAbs

To optimize the PEG chain length (PEG2K or PEG5K) and the BsAbs to mPEG molar ratio for tumor targeting, the DIO-loaded L*_sb_*MDDs (2K or 5K), DIO-loaded DNS-L*_sb_*MDDs (2K or 5K), and DIO-loaded HER2-L*_sb_*MDDs (2K or 5K) were prepared as follows: two BsAbs (anti-mPEG/anti-HER2 and anti-mPEG/anti-DNS) at various BsAbs/mPEG molar ratios (of 0.001:1, 0.002:1, 0.01:1, and 0.02:1) were separately mixed with a fixed amount of the DIO-loaded L*_sb_*MDDs(2K or 5K) in BSA/PBS buffer (0.05% w/v) for 1 h to form the DIO-loaded HER2-L*_sb_*MDDs(2K or 5K) and DIO-loaded DNS-L*_sb_*MDDs(2K or 5K), respectively. The HER2-overexpressing BC cell line of MCF-7/HER2 was seeded onto 24-well plates at a density of 5 × 10^4^ cells/well. The DIO-loaded BsAbs-L*_sb_*MDDs(2K or 5K) was added to the wells and incubated for 4 h. After removing the unbound NC, the cells were collected, washed, and resuspended in the PBS. Cellular uptake of the L*_sb_*MDDs, DNS-L*_sb_*MDDs, and HER2-L*_sb_*MDDs by the MCF-7/HER2 cells was quantitatively evaluated by flow cytometry. The presence of the DIO detected by excitation at 484 nm and emission at 501 nm was used as an indicator of the uptake amount of NCs.

### 2.9. Drug release of the optimal L*_sb_*MDDs(5K) and BsAbs-L*_sb_*MDDs(5K)

Amounts of drug released from Tynen^®^ (solvent-based DTX), the L*_sb_*MDDs, DNS-L*_sb_*MDDs, and HER2-L*_sb_*MDDs were investigated in PBS (containing 0.5% Tween 80) by a dialysis method. Briefly, 1 mL of the three L*_sb_*MDDs solutions containing 0.25 mg DTX or 0.25 mg/mL Tynen^®^ was loaded into a dialysis bag (MWCO 6000, Cellu-Sep^®^ T1, Orange Scientific, Seguin, TX) against 25 mL of release medium with shaking at a speed of 100 rpm at 37 °C. At a predetermined time point, the release medium in the dialysis bag was replaced with a fresh medium to maintain the sink conditions. The drug concentration was analyzed by the HPLC method described above. All measurements were carried out in triplicate.

### 2.10. Cell viability

Cell viabilities of the three BC cell lines of MCF-7, MCF-7/HER2, and SKBR-3 treated with Tynen^®^, the L*_sb_*MDDs, DNS-L*_sb_*MDDs, and HER2-L*_sb_*MDDs were evaluated by an MTT assay. Cells were seeded at a density of 5 × 10^4^ cells per well in 24-well plates and incubated for 24 h at 37 °C with 5% CO_2_. Then, they were treated with different concentrations of the formulations for 4 h under the same conditions. After incubation for 48 h, 50 μL of MTT (6 mg/mL) was added to each well for 3 h. The medium was removed and 200 μL of DMSO was added to each well and gently shaken to dissolve any purple formazan crystal formations. The absorbance of each well was measured at 550 nm (BioTek, Winooski, VT).

### 2.11. *In vitro* cellular uptake of the optimal L*_sb_*MDDs(5K) and elucidation of the cellular uptake mechanism

BC cell lines (MCF-7, MCF-7/HER2, and SKBR-3 cells) were seeded at a density of 5 × 10^4^ cells per well on 12-well microplates. The DIO-loaded L*_sb_*MDDs, DIO-loaded DNS-L*_sb_*MDDs, and DIO-loaded HER2-L*_sb_*MDDs (with a molar ratio of BsAbs to mPEG of 0.01:1) were added to separate wells and incubated for 0.5, 2, and 8 h. After incubation for different times, cells were collected, and analyzed by flow cytometry quantitatively evaluate the intracellular uptake of the L*_sb_*MDDs and the BsAbs-L*_sb_*MDDs by MCF-7, MCF-7/HER2, and SKBR-3 cells. To further understand the cellular uptake mechanism of the HER2-L*_sb_*MDDs by MCF-7/HER2 cells, cells were incubated for 60 min separately with cytochalasin D (10 μg/mL) as an inhibitor of phagocytosis and micropinocytosis (Kuhn et al., 2014), with amiloride (50 μM) as an inhibitor of micropinocytosis (Dutta & Donaldson, 2012), with methyl-β-cyclodextrin (MBCD, 0.5 mM) as an inhibitor of lipid rafts involved in caveolae-mediated endocytosis (Itoh et al., 2008), with nystatin (50 μg/mL) as an inhibitor of caveolae-mediated endocytosis (Kuhn et al., 2014), with chlorpromazine (20 μg/mL), sucrose (450 mM), or dynasore (40 μM) as inhibitors of clathrin-mediated endocytosis (Sahay et al., 2010), and with herceptin (0.5 μg/mL) as an inhibitor of the HER2 receptor, and then were treated with formulations in the presence of the inhibitors for 2 h. After incubation, cells were treated as aforementioned. The fluorescence was measured using flow cytometry (SA3800, Sony, San Jose, CA).

### 2.12. Intracellular localization of DIO-loaded -L*_sb_*MDDs

MCF-7/HER2 cells were seeded in 3.5-cm glass bottom dishes for 24 h. After 2 h of treatment with the DIO-loaded L*_sb_*MDDs, DNS-L*_sb_*MDDs, or HER2-L*_sb_*MDDs formulation, cells were stained with LysoTracker Red DND-99 for 30 min and Hoechst for 10 min to indicate lysosomes and nuclei, respectively. Subcellular localization of each target signal was observed using the TCS SP5 Confocal Spectral Microscope Imaging System (Leica, Wetzlar, Germany).

### 2.13. *In vivo* PK studies of intravenous administration

SD rats at 8 ∼ 10 weeks old were used to study the PK profiles of DTX after administration of Tynen^®^ (solvent-based DTX), the L*_sb_*MDDs, DNS-L*_sb_*MDDs, and HER2-L*_sb_*MDDs. Rats were given a single dosage of 8 mg/kg of each formulation *via* a jugular vein injection (three rats per group). Blood was collected from the jugular vein in heparinized tubes at 0.083, 0.25, 0.5, 1, 2, 4, 6, 8, 10, 24, 48, and 72 h after administration. Blood samples were centrifuged at 3000 rpm for 10 min to obtain plasma and were stored at −80 °C prior to analysis by UPLC/MS/MS (detailed describe in Supplemental information). In order to examine the *in vivo* binding activity of HER2-L*_sb_*MDD, a supplemental PK study was conducted to measure the HER2 binding activity of HER2-L*_sb_*MDD along with the detection of plasma DTX concentration at the same predetermined time points. The HER2 binding activity of HER2-L*_sb_*MDD in this PK study was detected by a cell-based ELISA method and the details were described in Supplemental information.

### 2.14. Tumor inhibition studies

All female nu/nu mice received a subcutaneous injection of 100 μL (containing 5 × 10^6^ cells) of the MCF-7/HER2 cell suspension in Matrigel into their right thighs of mice. Tumor growth was promoted by subcutaneously injecting 20 μg of estradiol valerate in 50 μL of sesame oil once a week near the neck. These tumor-bearing mice with around 200 mm^3^ tumor volumes were randomized into five groups: one control group (PBS) and four groups including Tynen^®^, the L*_sb_*MDDs, DNS-L*_sb_*MDDs, and HER2-L*_sb_*MDDs (5 mg DTX/kg, *n* = 6). Each formulation was injected once every 3 d for 12 d. The tumor volume was calculated by the modified ellipsoidal formula of 1/2 length × width^2^. Mice body weights and tumor volumes were measured every 3 d after the injection. The mice were sacrificed by CO_2_ and the tumors were harvested and weighed on day 21. The tumor inhibitory rate (%) was calculated as follows: (Wc − Wt)/Wc, where Wc is the tumor weight of the control group and Wt is the tumor weight of each formulation group.

### 2.15. *In vivo* biodistribution studies

The biodistribution study was evaluated in the MCF-7/HER2-bearing nu/nu mice. After tumor sizes had reached ∼200 mm^3^, Tynen^®^, the L*_sb_*MDDs, DNS-L*_sb_*MDDs, and HER2-L*_sb_*MDDs were administered at 40 mg DTX/kg through the tail vein. At the time points of 2 and 16 h, the mice were sacrificed by CO_2_ and perfused with a PBS buffer containing a 0.1% heparin solution to remove blood from the organs. The heart, liver, spleen, lung, kidney, and tumor were harvested. All organs were weighed and stored at −80 °C until being assayed for DTX. Organ extraction used a homogenization method. Briefly, a 5-fold volume of a PBS/0.1% heparin solution was added to each of the weighed tissues, and an SH-100 homogenizer (Kurabo Industries, Osaka, Japan) was used to homogenize the sample. The tissue homogenate (200 µL) was obtained, and the DTX concentration in the tissue was analyzed using the UPLC/MS/MS as the disposal method for plasma samples.

### 2.16. *In vivo* imaging of tumor-bearing mice

To compare the tumor-targeting efficiency of the HER2-L*_sb_*MDDs with those of the L*_sb_*MDDs and DNS-L*_sb_*MDDs, the DIR, a near-infrared fluorescent probe, was encapsulated into formulations, and then 200 µg/kg of the DIR was administered to mice *via* the tail vein. The mice were then monitored at 2, 8, and 24 h after the injection using an IVIS 200 Imaging System with an ICG emission filter (Perkin Elmer, Waltham, MA). For *ex vivo* imaging, the major organs of the heart, liver, spleen, lung, and kidney, as well as the tumor, were excised at 24 h post-injection.

### 2.17. Statistical analysis

Data are presented as the mean ± standard deviation (SD) of each group. The significance among samples was performed using a one-way analysis of variance (ANOVA). Significant differences between groups were indicated by *p < .05, **p < .01, and ***p < .001.

## 3. Results

### 3.1. Physical characterization of the L*_sb_*MDDs

To introduce mPEGylation to robust and previously developed promising delivery systems known as the L*_sb_*MDDs (Chen et al., 2015; Chen et al., 2016), the thin film of self-assembling micelles was hydrated with a lecithin/DSPE-PEG (2K or 5K) nanosuspension in this study. The micellar core of the so-obtained L*_sb_*MDDs was composed of DTX and DSPE-PEG2K, while the lipid shell consisted of lecithin and DSPE-PEG (2K or 5K) at a ratio of 40:15 (w/w). The average particle size and distribution pattern of the L*_sb_*MDDs(2K) and L*_sb_*MDDs(5K) are as shown in Figure 1(B,C), respectively. Results showed that the average particle sizes of the L*_sb_*MDDs(2K) and L*_sb_*MDDs(5K) were 131.0 ± 1.9 and 152.5 ± 3.28 nm, polydispersity index (PDIs) were 0.29 ± 0.067 and 0.25 ± 0.01, EE were 91.3 and 95%, ZPs were −38.4 ± 0.2 and −34.2 ± 0.1 mV, and DL were 5.93 and 6.16%, respectively. The structures of both the L*_sb_*MDDs formulations as observed in the TEM images (Figure 1(D,E), respectively) exhibited a spherical morphology and were well dispersed and separated.

### 3.2. Optimal modification of the L*_sb_*MDDs withanti-mPEG/anti-HER2 BsAbs *via* non-covalent binding

**Figure 2.**
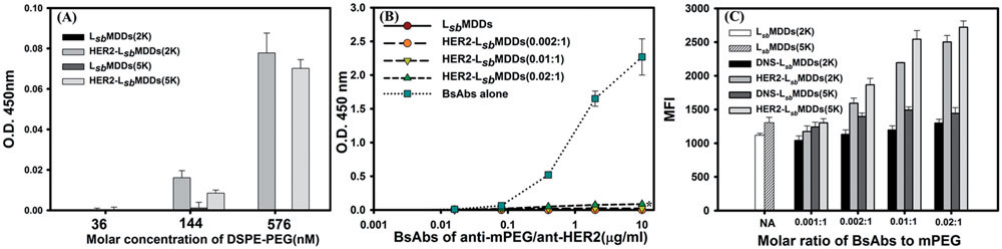
(A) Detection of BsAbs on HER2-L*_sb_*MDDs(2K or 5K) by a sandwich ELISA method (*n* = 3). (B) The optimal molar ratio of BsAbs to mPEG5K on the L*_sb_*MDDs(5K) was assessed at three different molar ratios of BsAbs to mPEG5K (of 0.002:1, 0.01:1, and 0.02:1) by an ELISA method (*n* = 3). *p < .05 compared to the L*_sb_*MDDs(0.01:1). **(C)** Cellular uptake of the L*_sb_*MDDs(2K and 5K), DNS-L*_sb_*MDDs(2K and 5K), and HER2-L*_sb_*MDDs(2K and 5K) with various molar ratios of BsAbs to mPEG (of 0.001:1, 0.002:1, 0.01:1, and 0.02:1) were measured by flow cytometry (*n* = 3).

To investigate whether anti-mPEG/anti-HER2 BsAbs could be non-covalently bound to the L*_sb_*MDDs(2 K or 5 K), the BsAbs non-covalently bound to HER2-L*_sb_*MDDs(2 K or 5 K) were detected by a sandwich ELISA. The anti-PEG backbone mAbs (AGP4) which can specifically bind to the backbone of the PEG chain were coated in 96-well plates to capture L*_sb_*MDDs(2 K or 5 K) with or without BsAbs modification. The HRP-conjugated secondary antibody was added for detecting the BsAbs on L*_sb_*MDDs and HRP activity was determined by absorbance at 405 nm (OD value) due to the oxidation product of ABTS. The results in Figure 2(A) demonstrate that the binding concentration of HER2-L*_sb_*MDDs(2 K or 5 K) in the formulations increased leading to an increase in the resulting absorbance, indicating that BsAbs were non-covalently bound to L*_sb_*MDDs with different chain length of mPEG (2 K or 5 K) followed with a concentration-dependent manner at a BsAbs/mPEG molar ratio of 0.01:1.

To determine the optimal ratio of the BsAbs (anti-mPEG/anti-HER2) to mPEG5K on the L*_sb_*MDDs, three HER2-L*_sb_*MDDs(5K) formulations prepared by mixing BsAbs at three different molar ratios of BsAbs to the mPEG-5K (of 0.002:1, 0.01:1, and 0.02:1) with a fixed amount of the L*_sb_*MDDs(5K). Then, all formulations were diluted to the indicated concentrations of BsAbs, and were added to the 96-well plates coated with the mPEG5K ligand (with a methoxy end group in the PEG chain) to assess the extent of unbound BsAbs to the L*_sb_*MDDs. Figure 2(B) shows that no absorbance with the molar ratios of BsAbs to mPEG of 0.002:1 and 0.01:1 was observed, indicating that all BsAbs were bound to the L*_sb_*MDDs with no detection of free BsAbs in the formulation. However, unbound BsAbs were detected in the higher BsAbs to mPEG molar ratio of 0.02:1. Further, no free BsAbs were detected when only the L*_sb_*MDDs was added as the negative control, whereas free BsAbs were detected at a concentration proportional to the amount of free BsAbs added as the positive control. This confirms that BsAbs could homogeneously non-covalent bind to the L*_sb_*MDDs with optimal molar ratios of BsAbs to mPEG-5K of 0.0 1 ∼ 0.02:1. The number of lipids in a 100 nm size liposome is about 80047 (Dennison et al., 2009; Mikhalin et al., 2014). At a molar ratio of lecithin: DSPE-PEG5K equal to 95:5, it is accordingly expected to have approximately 4002 molecules of DSPE-PEG5K molecules on the surface of the L*_sb_*MDDs with a similar size. Since the optimal ratio of BsAbs/mPEG was 0.01:1, there expected to have 40 molecules of BsAbs on one NC of L*_sb_*MDDs.

### 3.3. Optimization of tumor-targeting by BsAbsnon-covalently bound to the L*_sb_*MDDs

First, the bi-functional binding of the BsAbs (anti-mPEG/anti-HER2 and anti-mPEG/anti-DNS) for HER2-positive (MCF-7/HER2 and SKBR-3) and HER2-negative (MCF-7) cancer cells were examined and Trastuzumab was used as a positive control. Result of flow cytometry (Supplemental information Figure S1) showed that anti-mPEG/anti-HER2 BsAbs displays the binding activity to HER2 overexpressed cells of MCF-7/HER2 and SKBR-3 with the expression level of HER2 being greater for the former than the latter. Further, anti-mPEG/anti-HER2 BsAbs displays the similar results as that for Trastuzumab since the anti-HER2 portion of the former was constructed as a scFv according to a humanized anti-HER2 Ab (Trastuzumab). On the contrary, isotype BsAbs (anti-mPEG/anti-DNS) did not bind to any of three cancer cell lines examined in this study (MCF-7/HER2, SKBR-3, and MCF-7) since all three cancer cells did not express the ligand of DNS for binding.

Further, based on the uptake amount of the DIO-loaded L*_sb_*MDDs non-covalently bound with the BsAbs by the MCF-7/HER2, the optimal ratio of the two BsAbs (anti-mPEG/anti-DNS or anti-HER2) bound to the L*_sb_*MDDs composed of either mPEG2K or mPEG5K was examined, and referred to as the DNS-L*_sb_*MDDs(2K or 5K) and HER2-L*_sb_*MDDs(2K or 5K). Results in Figure 2(C) demonstrate that compared to the L*_sb_*MDDs, uptake amounts of both the HER2-L*_sb_*MDDs(2K) and the HER2-L*_sb_*MDDs(5K) increased with an increasing BsAbs to mPEG molar ratio, showing that the 0.01:1 ratio was optimal. Further, the uptake amount of the HER2-L*_sb_*MDDs(5K) at its optimal ratio was better than that of the HER2-L*_sb_*MDDs(2K). Contrarily, the uptake amounts of neither the L*_sb_*MDDs(2K or 5K) nor the DNS-L*_sb_*MDDs(2K or 5K) increased with an increasing BsAbs to mPEG molar ratio and were maintained at a similar uptake amounts as that for the L*_sb_*MDDs. This confirms that the HER2-L*_sb_*MDDs non-covalently bound of HER2 targeting BsAbs to the L*_sb_*MDDs was able to enhance the targetability to the cells that over-expressed HER2 on its cell membranes, resulting in an increase in cellular uptake, whereas those without the tumor targeting ligands (L*_sb_*MDDs) or with a non-expressing targeting ligand of DNS (DNS-L*_sb_*MDDs), were unable to enhance the cellular uptake. It was also concluded that the HER2-L*_sb_*MDDs(5K) incorporated the DSPE-PEG5K in the lipid shell of the L*_sb_*MDDs was more appropriate and was thus selected for subsequent studies with the abbreviated name of the HER2-L*_sb_*MDDs.

### 3.4. Physical characterizations of the optimal BsAbs-L*_sb_*MDDs

**Figure 3.**
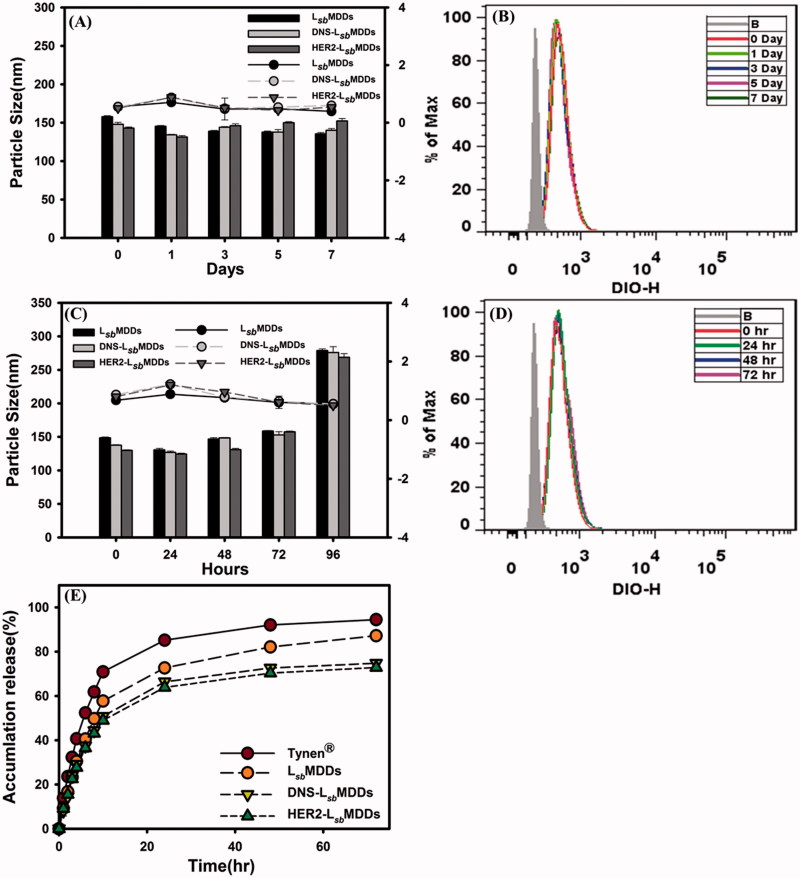
(A) The particles size of the L*_sb_*MDDs, DNS-L*_sb_*MDDs, and HER2-L*_sb_*MDDs during storage in PBS. (B) The binding activity of HER2-L*_sb_*MDDs during storage in PBS. (C) The particles size of the L*_sb_*MDDs, DNS-L*_sb_*MDDs, and HER2-L*_sb_*MDDs during storage in FBS. (D) The binding activity of HER2-L*_sb_*MDDs during storage in FBS. (E) Drug release profiles of Tynen^®^, the L*_sb_*MDDs, DNS-L*_sb_*MDDs, and HER2-L*_sb_*MDDs.

Physical characteristics were examined, including the average particle size, PDI, ZPs, and the storage stability of the optimal BsAbs-L*_sb_*MDDs composed of DSPE-PEG5K. Results showed that the resultant DNS-L*_sb_*MDDs and HER2-L*_sb_*MDDs were 148.4 ± 1.04 and 152.3 ± 0.94 nm in size, respectively, which were similar to the L*_sb_*MDDs alone (152.5 ± 2.88 nm; *p >* .05). The PDI values for all the particles were around 0.25. These results indicated that the attachment of both the BsAbs which were non-covalently bound to the L*_sb_*MDDs caused no significant change in the average particle size or distribution. Respective values of the ZPs of the DNS-L*_sb_*MDDs and HER2-L*_sb_*MDDs were −40.5 ± 0.62 and −40.1 ± 0.62 mA, which were more negative than that of the L*_sb_*MDDs. In another report, the ZPs consistently increased after BsAbs were incorporated into NPs (Gao et al., 2011). To examine the *in vitro* stability of the L*_sb_*MDDs, DNS-L*_sb_*MDDs, and HER2-L*_sb_*MDDs, they were incubated in PBS and FBS to monitor changes in the average particle sizes and PDI with respect to time, and the preservation of HER2 binding activity was determined as well. The results are as shown in Figure 3(A) that the average particle size and PDI of both BsAbs-L*_sb_*MDDs formulations were stable for at least 7 d in PBS, with no precipitation or significant changes observed. However, Figure 3(C) shows both BsAbs-L*_sb_*MDDs formulations incubated in FBS were observed to be stable for only 72 h with no significant changes in the average particle size or PDI. The particle size increased to >200 nm which means that the NPs became aggregated, and precipitation of the drug was observed at 96 h. To determine the HER2 binding activity, the cellular uptake of DIO-loaded HER2-L*_sb_*MDDs after different incubation time in PBS and FBS was examined on MCF7/HER2 which overexpressed HER2. Figure 3(B,D) shows that the similar extent of cellular uptake were observed for all samples compared to that at 0 h even after 7 d in the presence of PBS and 72 h in FBS indicating that HER2-L*_sb_*MDDs retained the same ability to bind to MCF-7/HER2 cells. These results indicate that BsAbs modified L*_sb_*MDDs was stable for at least 72 h under physiological conditions.

### 3.5. *In vitro* release profiles of DTX from the optimal BsAbs-L*_sb_*MDDs

Drug release profiles were calculated by plotting the release percentage of the drug from the optimal DTX-loaded BsAbs-L*_sb_*MDDs when compared to that for the solvent-based formulation (Tynen^®^), and the DTX-loaded L*_sb_*MDDs. Results in Figure 3(E) illustrate that the initial release of the DTX from Tynen^®^ was the fastest among all the formulations examined with 80% of the DTX being released within 24 h. The release rate of the DTX from the L*_sb_*MDDs formulations was slower than that for Tynen^®^ with 70% being released within 24 h. The release rates for both the BsAbs-L*_sb_*MDDs formulations were found to be similar and the slowest, with 60% being released in a 24 h period. These results indicated that a greater proportion of the DTX was entrapped in the micellar core of the L*_sb_*MDDs and BsAbs -L*_sb_*MDDs. The shielding effect of the non-covalently bound of both the BsAbs onto the outer shell of the L*_sb_*MDDs might have further impeded the diffusion of the DTX resulting in an even smaller portion of the DTX being released.

### 3.6. *In vitro* cytotoxicity

**Figure 4.**
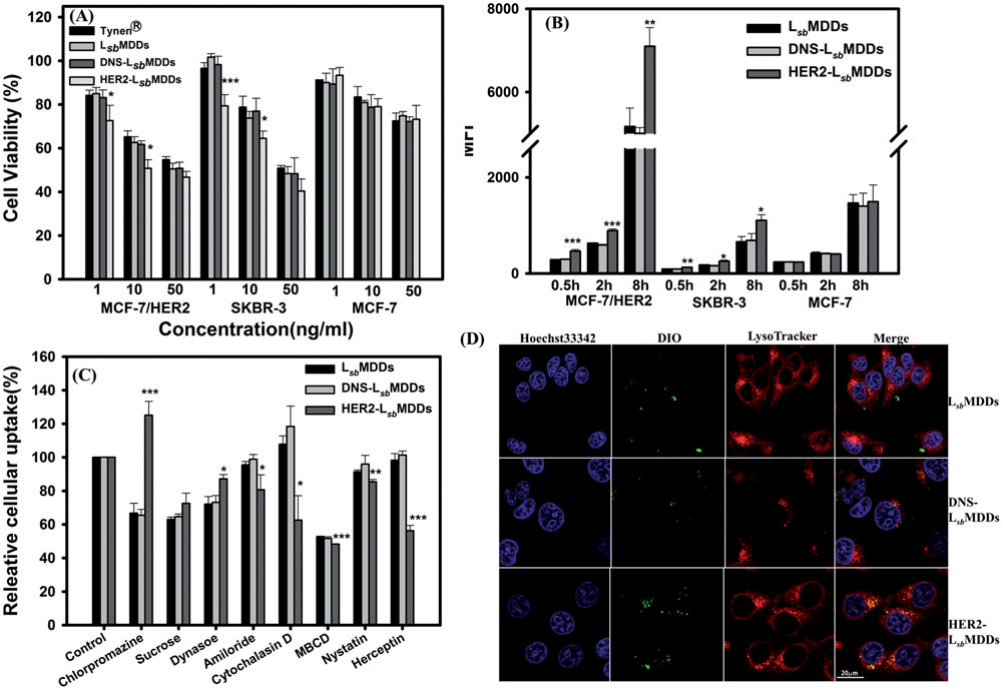
(A) Cell viabilities of Tynen^®^, the L*_sb_*MDDs, DNS-L*_sb_*MDDs, and HER2-L*_sb_*MDDs in the MCF-7/HER2, SKBR-3, and MCF-7 cell lines (*n*= 4). (B) Cellular uptake of the L*_sb_*MDDs, DNS-L*_sb_*MDDs, and HER2-L*_sb_*MDDs was examined by flow cytometry after incubating DIO-loaded formulations with the MCF-7/HER2, SKBR-3, and MCF-7 cell lines at the time points of 0.5, 2, and 8 h (*n*= 3). (C) Cells were treated with 5 μM of the DIO-loaded L*_sb_*MDDs for 2 h in the presence of various inhibitors. Uptake is presented as the percentage of the control. (D) Confocal images of MCF-7/HER2 cells after treatment with the L*_sb_*MDDs, DNS-L*_sb_*MDDs, and HER2-L*_sb_*MDDs for 2 h after triple fluorescence-labeling experiments: red fluorescence from LysoTracker, green fluorescence from DIO, and blue fluorescence from Hoechst 33342 for nuclei. Colocalization of red and green fluorescence was observed in cells (scale bar =20 μm). *p < .05, **p < .01, and ***p < .001 compared to the L*_sb_*MDDs.

The cytotoxicity of Tynen^®^, the L*_sb_*MDDs, and BsAbs-L*_sb_*MDDs against the HER2-positive (MCF-7/HER2 and SKBR-3) and HER2-negative (MCF-7) cancer cells was examined. Result in Figures 4(A) and S2 (Supplemental information) shows that a dose-dependent effect is observed with all the formulations on HER2^+^ cells lines but not on the MCF-7 cells. The reason is that MCF-7 cells are less sensitive to DTX (the IC_50_ of MCF-7/HER2 is 52 ng/mL, while that of the MCF-7 is 763 ng/mL) resulting that a low dose range of DTX did not cause a significant difference in cell cytotoxicity. Both the L*_sb_*MDDs and DNS-L*_sb_*MDDs formulations exhibited similar cytotoxicity to that of Tynen^®^ against the three cell lines (MCF-7/HER2, SKBR3, and MCF-7). Indeed, the HER2-L*_sb_*MDDs produced a significantly higher cytotoxicity to the MCF-7/HER2 and SKBR-3 cancer cell lines, both of which overexpressed HER2 on cell membranes, but displayed similar cytotoxicity to MCF-7 (HER2^-^) which poorly expresses HER2 as those for Tynen^®^, the L*_sb_*MDDs, and DNS-L*_sb_*MDDs. This demonstrates that the increase in cytotoxicity induced by the HER2-L*_sb_*MDDs requires the presence of the HER2 tumor antigen. It was concluded that the BsAbs confirmed the tumor specificity and enhanced the cytotoxicity of the L*_sb_*MDDs toward antigen-positive cancer cells.

### 3.7. Cellular uptake of the optimal BsAbs-L*_sb_*MDDs

To examine the targetability of the optimal BsAbs-L*_sb_*MDDs to HER2-positive tumor cell lines, the cellular uptake of the DIO-loaded BsAbs-L*_sb_*MDDs was examined after incubating the DIO-loaded BsAbs-L*_sb_*MDDs at a 0.01:1 molar ratio of BsAbs to mPEG with the HER2-positive cell lines of MCF-7/HER2 and SKBR-3 and the HER2-negative cell line of MCF-7 at 37 °C. As shown in Figure 4(B), cellular uptake amount of the HER2-L*_sb_*MDDs by the MCF-7/HER2 and SKBR-3 cells at all three time points (0.5, 2, and 8 h) were higher than those for the L*_sb_*MDDs and DNS-L*_sb_*MDDs, both of which had the same level of cellular uptake at all-time points in the three cell lines examined. Figure 4(B) also illustrates that uptake of the HER2-L*_sb_*MDDs was the same as those for the L*_sb_*MDDs and DNS-L*_sb_*MDDs in the antigen-negative MCF-7 cell line. This further confirmed that the optimal HER2-L*_sb_*MDDs non-covalently bound of anti-HER2/anti-mPEG BsAbs was able to enhance the cellular uptake by MCF-7/HER2 and SKBR-3 cells, both of which over-expressed HER2 on their cell membranes.

### 3.8. Cellular uptake mechanism

The general pathways of NCs internalized into cells are known to be phagocytosis, macropinocytosis, caveolae-dependent, and clathrin-mediated endocytosis (Zhao et al., 2011). Herein, we used cytochalasin D as a phagocytosis and macropinocytosis inhibitor, amiloride as a macropinocytosis inhibitor, MBCD as an inhibitor of lipid rafts involved in caveolae-dependent endocytosis, nystatin as a caveolae-dependent endocytosis inhibitor, chlorpromazine, sucrose, and dynasore as clathrin-mediated endocytosis inhibitors, and herceptin as a HER2 blocker, to determine which pathway participates in HER2-mediated cellular uptake. Results in Figure 4(C) show that the fluorescence intensities in the chlorpromazine, sucrose, and dynasore-treated L*_sb_*MDDs and DNS-L*_sb_*MDDs were significantly reduced when compared to that in the HER2-L*_sb_*MDDs. On the other hand, the relative uptake levels of the HER2-L*_sb_*MDDs in the presence of amiloride, cytochalasin D MBCD, and nystatin were considerably lower than those of the L*_sb_*MDDs in the MCF-7/HER2 cells. Results also show that the fluorescence intensity was notably reduced by the herceptin treatment in the HER2-L*_sb_*MDDs, but had no effect on the other two groups. These results demonstrate that the caveolae-mediated endocytosis and the macropinocytosis were more important for the cellular uptake of the HER2-L*_sb_*MDDs than for the L*_sb_*MDDs.

Promotion of the internalization of the L*_sb_*MDDs into tumor cells by BsAbs of HER2 was assessed. Confocal microscopic graphs in Figure 4(D) visualize green fluorescence from the DIO-loaded L*_sb_*MDDs, red fluorescence from the LysoTracker for lysosomes, and nuclear DNA labeled with Hoechst 33342 which emits blue fluorescence. The red and green fluorescence signals were imaged as being colocalized in MCF-7/HER2 cells treated with the HER2-L*_sb_*MDDs for 2 h, which demonstrated that anti-mPEG/anti-HER2 BsAbs could specifically deliver the HR2-L*_sb_*MDDs to MCF-7/HER2 cells, and when accompanied by HER2-L*_sb_*MDDs treatment, markedly increased colocalization of lysosomes with NCs of the HER2-L*_sb_*MDDs. It was concluded that anti-mPEG/anti-HER2 BsAbs can mediate selective binding and internalization of the HER2-L*_sb_*MDDs into the HER2^+^ cancer cells.

### 3.9. PK studies of the optimal BsAbs-L*_sb_*MDDs

**Figure 5.**
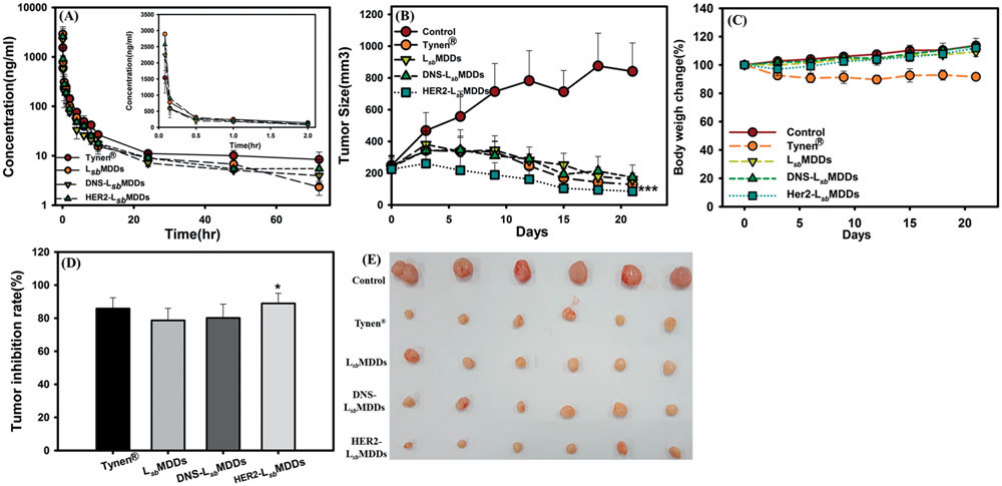
(A) Plasma concentration-time curves of docetaxel after intravenous administration of Tynen^®^, the L*_sb_*MDDs, DNS-L*_sb_*MDDs, and HER2-L*_sb_*MDDs at a dose of 8 mg/kg to rats (*n*= 3). (B) The MCF-7/HER2 tumor growth curve after intravenous administration at a dosing regimen of 5 mg/kg Q3D*4 (*n*= 6). (C) Body weight changes in tumor-bearing mice. (D) Tumor inhibition rates and (E) photographs showing the size of tumors after tumor-bearing mice were sacrificed on day 21. *p < .05 and ***p < .001 compared to the L*_sb_*MDDs on day 21.

PK profiles of the BsAbs-L*_sb_*MDDs were performed and compared to those with the L*_sb_*MDDs alone and Tynen^®^. The related PK parameters estimated by WinNonlin are listed in Supplemental information Table S1. All the PK profiles plotted in Figure 5(A) show a high initial DTX concentration after the injection, followed by a rapid decline to the terminal phase which gradually reached a steady-state concentration, and which was observed to be slightly higher for Tynen^®^ than the other three formulations. Supplemental information Table S1 illustrates that a 3 ∼ 6-fold higher initial concentrations (C_0_) for the three L*_sb_*MDDs formulations were observed as compared to that for Tynen^®^. The AUC_0–72_ and AUC_0–inf_ values for the L*_sb_*MDDs formulations were similar to those of Tynen^®^. The AUC_0–inf_ for L*_sb_*MDDs (2276 ± 473 h*ng/mL) was slightly higher than those of the DNS-L*_sb_*MDDs and the HER2-L*_sb_*MDDs; nevertheless, the difference between them was insignificant (*p* > .05). CL and V values of the HER2-L*_sb_*MDDs were 1.08- and 1.2-times larger than those of the L*_sb_*MDDs, while there was no dramatic difference in the half-life of these three different L*_sb_*MDDs formulations. In a supplemental PK study, the HER2 binding activity of HER2-L*_sb_*MDDs along with the detection of plasma DTX concentration at the same predetermined time points was measured and results are shown in Figure S3 (in Supplemental information). As shown in Figure S3, the plasma PK profile of DTX after administration of HER2-L*_sb_*MDDs was similar to that revealed in Figure 5(A) confirming the reproducibility of the PK study. Along with the plasma DTX concentration illustrated by Figure S3 is the binding activity of HER2-L*_sb_*MDDs remained in the plasma at each time point. Since it has been confirmed that after administration of DTX-loaded L*_sb_*MDDs, most of DTX in the plasma was encapsulated in L*_sb_*MDDs (Sheu et al., 2017). Therefore, the binding activity of HER2-L*_sb_*MDDs that encapsulated DTX was measured at the same DTX concentration (all were diluted to 5 ng/mL DTX) as an indication of the same HER2-L*_sb_*MDDs concentration being loaded in the measurement of the binding activity. Using the binding amount of HER2-L*_sb_*MDDs at time point of 1 h as 100%, the binding activity expressed as percentage remained for each time point as compared to that at 1 h is illustrated in Figure S3. The results demonstrated that the binding activity of HER2-L*_sb_*MDDs remained in the plasma gradually decreased but maintained at a 50% binding activity at 72 h. The gradual loss of the binding activity of HER2-L*_sb_*MDDs means that the BsAbs was detached from HER2-L*_sb_*MDDs leading to the less amount of NCs being able to bind to HER2 receptor. Nevertheless, the 50% binding activity of DTX-loaded HER2-L*_sb_*MDDs remained in plasma was still stable and targetable even being subjected to vigorous blood flow for 72 h.

### 3.10. *In vivo* antitumor efficacy of the HER2-L*_sb_*MDDs in tumor-bearing mice

The anti-tumor effects of Tynen^®^ and the BsAbs-L*_sb_*MDDs were evaluated in a HER2-positive cancer cell (MCF-7/HER2) model. Results are shown in Figure 5(B,E) clearly demonstrate that the three L*_sb_*MDDs formulations and Tynen^®^ all efficaciously inhibited the growth of MCF-7/HER2 tumors after treatment. The HER2-L*_sb_*MDDs showed the greatest anti-tumor effect among all the formulations in the MCF-7/HER2 tumor-bearing mice (Figure 5(B)). Tumor growth in the HER2-L*_sb_*MDDs treatment group was significantly suppressed as compared to those of Tynen^®^ (*p <* .05 on day 21), the L*_sb_*MDDs alone (*p <* .001 on day 21), and the negative control of the DNS-L*_sb_*MDDs (*p <* .05 on day 21). The tumor inhibitory rate of the HER2-L*_sb_*MDDs was 88.9%, whereas they were 85.8, 78.4, and 80.1% for Tynen^®^, L*_sb_*MDDs, and DNS-L*_sb_*MDDs, respectively (Figure 5(D)). Nevertheless, the weight change profiles of all the treatments illustrated in Figure 5(C) demonstrate that there was greater weight loss in the Tynen^®^ treatment group than for any of the three L*_sb_*MDDs formulations, indicating that the three L*_sb_*MDDs formulations induced less systemic toxicity than did Tynen^®^. Thus, it was concluded that the treatment with the HER2-L*_sb_*MDDs was more efficacious in inhibiting tumor growth than all of the other formulations while showing no signs of adverse side-effects.

### 3.11. Biodistribution assessment of the HER2-L*_sb_*MDDs

**Figure 6.**
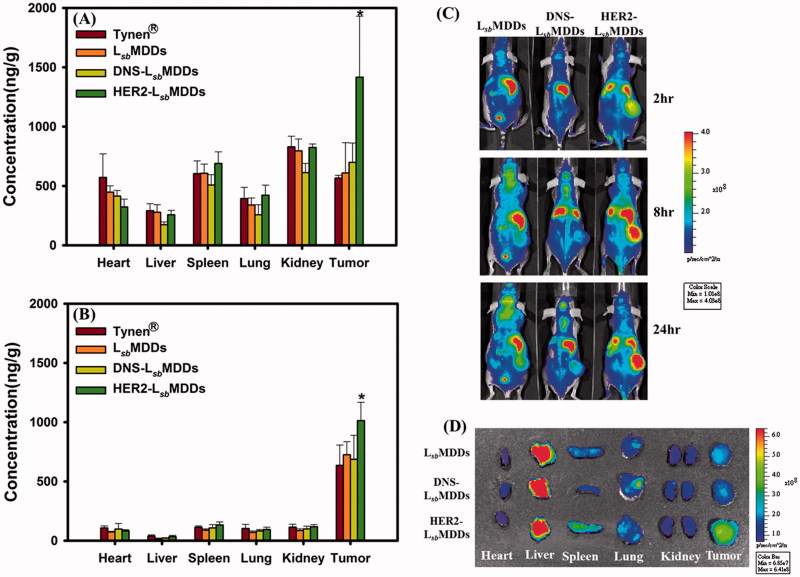
Tissue distributions of docetaxel (DTX) at (A) 2 and (B) 16 h after intravenous administration of Tynen^®^, the L*_sb_*MDDs, DNS-L*_sb_*MDDs, and HER2-L*_sb_*MDDs at a dose of 40 mg/kg into MCF-7/HER2 tumor-bearing nu/nu mice (*n = 3*). (C) Mice were imaged at 2, 8, and 24 h with an IVIS spectrum optical imaging system after being intravenously injected with the DIR-loaded L*_sb_*MDDs, DIR-loaded DNS-L*_sb_*MDDs, and DIR-loaded HER2-L*_sb_*MDDs (200 μg/kg of DIR). (D) *Ex vivo* fluorescence images of excised organs and tumors at 24 h post-injection of the L*_sb_*MDDs, DNS-L*_sb_*MDDs, and HER2-L*_sb_*MDDs in MCF-7/HER2 tumor-bearing nu/nu mice. *p < .05 compared to the L*_sb_*MDDs.

Biodistributions of the HER2-L*_sb_*MDDs in various organs after an IV administration were assessed compared to those for Tynen^®^, the L*_sb_*MDDs, and the DNS-L*_sb_*MDDs in MCF-7/HER2 tumor-bearing mice. At 2 and 16 h after the IV administration, organs, including the heart, liver, spleen, lung, and kidney, as well as the tumor were excised to analyze them for DTX. DTX concentrations in these tissues at 2 and 16 h are, respectively, shown in Figure 6(A,B). DTX was observed to be mainly distributed in the heart, spleen, kidney, and tumor at 2 h after administration, and a higher concentration of DTX was only retained in the tumor at 16 h for all the four formulations. At both time points, the DTX concentrations following an injection of Tynen^®^ were found to be slightly higher than those for the L*_sb_*MDDs and DNS-L*_sb_*MDDs formulations in all the tissues, except in the tumor tissues. Furthermore, a statistically significantly higher DTX concentration was only shown in tumor tissues for treatment at 2 and 16 h with the HER2-L*_sb_*MDDs respectively being 2.32 and 1.3-fold higher than the other three formulations. At 16 h after the injection, the DTX in the tumor site was still maintained at a 5 ∼ 10-fold higher concentration compared to that in other tissues for all the four formulations. These results indicated that the HER2-L*_sb_*MDDs not only enhanced the targeting to the tumor site resulting in a higher accumulation of DTX in the tumor, but also further retained the DTX in the tumor for a longer time to improve the therapeutic efficacy.

### 3.12. *In vivo* imaging of tumor-bearing mice

To investigate tumor targeting of the BsAbs-L*_sb_*MDDs *in vivo*, images of mice on an IVIS imaging system were captured at 2, 8, and 24 h post-injection of the DIR-loaded L*_sb_*MDDs and the DIR-loaded BsAbs-L*_sb_*MDDs. As shown in Figure 6(C), the fluorescent intensity (total flux) of the HER2-L*_sb_*MDDs was primarily found at tumor sites (except in liver) and was 2 ∼ 3-fold higher than that for the L*_sb_*MDDs and DNS-L*_sb_*MDDs at 24 h, indicating higher accumulation of the HER2-L*_sb_*MDDs in the tumor. Additionally, *ex vivo* images in Figure 6(D) from harvested tumors also confirmed obviously higher fluorescence in the HER2-L*_sb_*MDDs group than those from the L*_sb_*MDDs and DNS-L*_sb_*MDDs groups, which was further evidence of the higher tumor targeting efficiency of the HER2-L*_sb_*MDDs. These data indicated that BsAbs of anti-mPEG/anti-HER2 were able to actively target the HER2^+^ cancer cells, thereby facilitating enhanced HER2-L*_sb_*MDDs accumulation in HER2^+^ tumors to improve the therapeutic efficacy and minimize potential side effects as a result of a preferable biodistribution in tumor cells. Both *in vivo* images in Figure 6(C) and *ex vivo* images in Figure 6(D) illustrate that the fluorescent signal was the highest detected in the liver, which was in contrary to what was observed in biodistribution study that demonstrated the accumulation of DTX was higher in tumor than that in liver. This discrepancy might be explained by that the fluorescent dye uptake *via* NCs could be retained longer in the liver in comparison to DTX resulting in the accumulation of fluorescent dye in the liver with showing the highest signal.

## 4. Discussion

To enable active tumor targeting of NCs, we established non-covalently bound of BsAbs (anti-mPEG/anti-tumor) to mPEGylated NCs of L*_sb_*MDDs designated L*_sb_*MDDs(2K and 5K), which were based on chemotherapeutic drug-loaded polymeric micelles stabilized by a lipid layer (lecithin/DEPSE-PEG) during the self-assembling hydration of micelles. By taking advantage of the unique strengths of the lecithin-based mixed polymeric micelles (LMPMs) and liposomes, the supported lipid shell composed an appropriate ratio of lecithin and DSPE-PEG was fused onto the polymeric micellar core of the L*_sb_*MDDs by ultrasonication. The physical characteristics of the L*_sb_*MDDs were a mean size of <200 nm, an EE of >90%, and DL of >5%. The surface of the L*_sb_*MDDs modification by PEG chains provides a steric barrier to prevent the opsonization and evade the reticuloendothelial system (RES). The outer structure of the L*_sb_*MDDs consisted of lecithin and DSPE-PEG at molar ratios of 95/5. When the PEG contents in the formulations were 5%, the structure was brush-like (Allen et al., 2002), which enabled the BsAbs to be non-covalently bound to methoxy terminus of the straight PEG chain *via* the anti-mPEG Fab fragments of BsAbs, to enhance active targeting of the mPEGylated NCs to the tumors *via* the anti-tumor scFv fragment of the BsAbs.

Figure 2(A) shows that the BsAbs could non-covalently bound to both the L*_sb_*MDDs(2K) and L*_sb_*MDDs(5K) *via* the methoxy end group of the mPEG chain that linked to the DSPE located on the surface of the L*_sb_*MDDs(2K) or L*_sb_*MDDs(5K). Further, the molar ratio of BsAbs to mPEG5K on the L*_sb_*MDDs(5K) was optimized based on the extents of the free BsAbs observed at the three different molar ratios of BsAbs to the L*_sb_*MDDs(5K). As shown in Figure 2(B), no free BsAbs were detected when the molar ratio was 0.01:1, whereas some extent of the free BsAbs was observed at a higher molar ratio of 0.02:1. This confirmed that an optimal molar ratio of the BsAbs to L*_sb_*MDDs(5K) should be 0.01 ∼ 0.02:1.

Amounts of the mPEGylated L*_sb_*MDDs taken up with either the DSPE-PEG2K or the DSPE-PEG5K were compared after the BsAbs were non-covalently bound to the L*_sb_*MDDs. Results showed that the longer the PEG chain was, the higher the cellular uptake was observed. Contrary to our results, the study by Charmainne & Chithrani reported that the shorter PEG chain lengths (PEG2K vs. PEG5K) resulted in higher uptake for two grafting densities (Charmainne & Chithrani, 2014). It was attributed to that the shorter chain lengths have higher cancer cell uptake due to a greater probability of nonspecific protein adsorption, which mediates the entry of inorganic NPs by receptor-mediated endocytosis. Conforming to our results, the study on delivering small interfering (si)RNA with lipid-polymer hybrid NPs reported that the formulation with DSPE-PEG5K had higher tumor accumulation than did that with DSPE-PEG3K (Zhu et al., 2015). Sadzuka et al. also reported that the longer PEG chain length in the 1-monomethoxypolyethyleneglycol-2,3-dimyristoylglycerol(PEG-DMG) and 1-monomethoxypolyethyleneglycol-2,3-distearoylglycerol (DSG) groups increased tumor cell uptake of liposome, i.e. the value of PEG5K was better than that of PEG2K (Sadzuka et al., 2003). It was attributed to that a longer PEG chain more easily attached to the tumor cell membrane. Along with this, BsAbs non-covalently bound on the methoxy terminal end of longer PEG chains make BsAbs-L*_sb_*MDDs more easily attach to the tumor cells resulting in the increase of tumor cell uptake.

The isotype control of anti-mPEG/anti-DNS BsAbs non-covalently bound to the L*_sb_*MDDs did not alter the physical characteristics of the L*_sb_*MDDs or the non-specific targeting to the tumor sites. The ZPs of the L*_sb_*MDDs was observed to be negative, thus conferring the lower interactions with the plasma proteins than the positively charged ones, which are expected to strongly interact with the blood components causing a higher extent of the drug leakage (Tenzer et al., 2013). The ZPs of both the BsAbs-L*_sb_*MDDs formulations were even more negative, indicating that the BsAbs were bound to the termini of mPEG chains. Drug release profiles described in Figure 3(E) also shows that the BsAbs-L*_sb_*MDDs formulations released drugs more slowly than did the L*_sb_*MDDs, implying that the non-covalent binding of BsAbs to NCs might have retarded drug leakage from the NCs by providing a stabilization effect on the shell structure of the L*_sb_*MDDs and a shielding effect on the permeation of the DTX.

As shown in Figure 4(A), enhanced cytotoxicity induced by HER2-L*_sb_*MDDs was only observed for the HER2-presenting tumor cell lines of the MCF-7/HER2 and the SKBR-3. Contrarily, Tynen^®^ and those L*_sb_*MDDs formulations without the BsAbs bound or bound with the isotype control antibody of the anti-DNS expressed similar cytotoxicities to tumor cell lines regardless of whether or not they presented the corresponding antigen. The uptake amount of the DIO-loaded HER2-L*_sb_*MDDs as shown in Figure 4(B) at all-time points was obviously greater than that for the L*_sb_*MDDs without BsAbs bound or bound with the isotype control antibody of the anti-DNS in HER2 over-presenting tumor cells of MCF-7/HER2 and SKBR-3, but not in MCF-7 tumor cells poorly presenting HER2. The cellular uptake of the HER2-L*_sb_*MDDs is higher in MCF-7/HER2 than SKBR-3 cell lines, because the HER2 expressed at the surface of the MCF-7/HER2 is higher than the SKBR-3 cell lines (Supplemental information Figure S1). This further confirmed that BsAbs can selectively bind to a tumor and enhance the cytotoxicity of the BsAbs-L*_sb_*MDDs toward the antigen-positive cancer cells as a result of an increased amount taken up.

To understand the mechanism of the cellular uptake of the L*_sb_*MDDs, results in Figure 4(C), show that most clathrin-mediated endocytosis inhibitors effectively reduced L*_sb_*MDDs uptake in the MCF-7/HER2 cells. However, chlorpromazine treatment increased cellular uptake of the HER2-L*_sb_*MDDs, but reduced uptake of the L*_sb_*MDDs, and the DNS-L*_sb_*MDDs. It was reported that the HER2 expression can inhibit the downregulation by having a negative effect on the formation of the clathrin-coated structures (Cortese et al., 2013). Since the inhibitory property of chlorpromazine acts through its ability to translocate clathrin from the cell surface to the intracellular endosomes (Dutta & Donaldson, 2012), this effect may indirectly increase the HER2 expression on the cell surfaces, thus causing more HER2-L*_sb_*MDDs to be transported into the cells. These results also indicated that the clathrin-mediated endocytosis is a more-preferable pathway for the cellular uptake of the L*_sb_*MDDs. Similar results can also be seen in the dynasore- and sucrose-treated groups. Nonetheless, when compared to the chlorpromazine-treated groups, dynasore and sucrose still had inhibitory effects, even with the HER2-L*_sb_*MDDs. Dynasore inhibits the clathrin-mediated endocytosis by blocking the GTPase activity of dynamin (Macia et al., 2006). Since dynamin is involved in new vesicle formation on the membranes (Henley et al., 1999), it might more directly influence the HER2-mediated transport. Overall results shown in Figure 4 suggest that the macropinocytosis and caveolae-mediated endocytosis were more-preferable pathways for HER2-L*_sb_*MDDs uptake than for L*_sb_*MDDs uptake. Furthermore, the BsAbs-L*_sb_*MDDs was taken up by receptor-mediated endocytosis, so it might be able to avoid the particles being pumped out by the P-glycoprotein (P-gp) or the breast cancer resistance protein (BCRP) in multiple drug-resistant cells.

Figure 5(B) demonstrates that the HER2-L*_sb_*MDDs significantly enhanced the *in vivo* anticancer activity, as shown by the higher growth inhibition rate in the HER2-overexpressing tumor cells. This likely can be attributed to the increased cytotoxicity of the HER2-L*_sb_*MDDs against the tumor cells over-expressing the HER2 anti-tumor antigen as shown in Figure 4(A). Further, according to the biodistribution study and the IVIS images in Figure 6, the DTX concentration after an IV injection of the HER2-L*_sb_*MDDs was highest at the tumor site, but with a similar DTX concentration to those for Tynen^®^, the L*_sb_*MDDs, and DNS-L*_sb_*MDDs in other organs of the heart, liver, spleen, lung, and kidney. Overall, the enhanced chemotherapeutic efficacy of the HER2-L*_sb_*MDDs can be attributed to the preferable biodistribution to tumor sites by active targeting of the HER2-L*_sb_*MDDs endowed with non-covalently bound of BsAbs and the enhanced DTX uptake amount *via* an endocytosis-mediated pathway leading to a greater accumulation and a longer retention of the DTX in the tumor.

Active targeting strategies facilitate NC internalization, binding, and homing to targeted cells. Antibody-drug conjugates (ADCs) consisting of highly potent cytotoxic agents covalently linked to a mAb are an emerging novel class of chemotherapeutics. However, the most commonly identified weaknesses limiting the effective uses of ADCs are the low anticancer drug potency, low antigen selectivity, and unstable linkers (Perez et al., 2014). Antibody-NP conjugates have great benefits in overcoming limitations in current approaches as NPs have the ability to release a drug at desirable sites, improve cell penetration, and cross biological barriers by conjugating Abs with high affinity. However, the method for Ab conjugation to NCs has the same challenges. Also, chemical modifications cause heterogeneous orientations, Ab lose their functions and are non-reproducible, and site-directed modifications cause complex operations and high costs (wasting large amounts of Abs) (Haberger et al., 2014). The primary goal of conjugating targeted ligands to NCs is to not lose the functionality of the ligand targeting. Using humanized BsAbs non-covalently bound to the L*_sb_*MDDs avoids direct, potentially denaturing interactions with NC surfaces, minimizing possible alterations of NC properties, and low immunogenicity. Our results showed no obvious changes in the physical properties of the L*_sb_*MDDs with the non-covalently bound of BsAbs. They also confirmed that the BsAbs were bound to the ends of mPEG molecules, thus orienting the anti-tumor scFv portion of the BsAbs outward and minimizing steric masking of the BsAbs by mPEG. This could be a simple and promising way to introduce active tumor targetability to the drug-loaded mPEGylated NCs to enhance their chemotherapeutic efficacy and minimize the systemic toxicities.

## 5. Conclusions

In conclusion, a BsAbs-modified L*_sb_*MDDs was established which minimized the changes in physical properties and structures and conferred the BsAbs target specificity to high drug-loaded NCs of the L*_sb_*MDDs. The BsAbs-modified L*_sb_*MDDs was effectively taken up by a combination of both passive targeting by the EPR and active targeting by internalization. The BsAbs non-covalently bound to the L*_sb_*MDDs can target antigen-overexpressing tumors with a drug cargo to enhance the drug uptake and the accumulation in tumors, leading to greater antitumor activities against antigen-positive tumors. This well-characterized platform can be applied to the BsAbs targeting other tumor markers, such as the prostate-specific membrane antigen (PSMA), EGFR, VEGF, and programed death-ligand 1 (PD-L1), non-covalently bound to mPEGylated NCs that can be loaded with chemotherapeutic drugs including paclitaxel, irinotecan, rapamycin, etc.

## Supplementary Material

IDRD_Sheu_et_al_Supplemental_Content.docx
