# Supplementary material for: Bispecific antibodies (anti-mPEG/anti-HER2) for active tumor targeting of docetaxel (DTX)-loaded mPEGylated nanocarriers to enhance the chemotherapeutic efficacy of HER2-overexpressing tumors
Source: Drug Deliv. 2018 May 2;25(1):1066–79. doi: 10.1080/10717544.2018.1466936 (PMC6058516; doi:10.1080/10717544.2018.1466936)
Supplement: IDRD_Sheu_et_al_Supplemental_Content.docx [file IDRD_A_1466936_SM0587.docx]

**Bispecific Antibod****ies (Anti-mPEG/Anti-HER2) for Active Tumor Targeting of Docetaxel (DTX)-Loaded mPEGylated Nanocarriers to Enhance the Chemotherapeutic Efficacy of HER2-Overexpressing Tumors**

Chia-Yu Su^a#^, Michael Chen^b#^, Ling-Chun Chen^c^, Yuan-Soon Ho^d^, Hsiu-O Ho^a^, Shyr-Yi Lin^e,f,g^, Kuo-Hsiang Chuang^b,h*^, and Ming-Thau Sheu^a*^

^a^School of Pharmacy, College of Pharmacy, Taipei Medical University, Taipei, Taiwan, ROC

^b^Ph.D. Program in Clinical Drug Development of Chinese Herbal Medicine, Taipei Medical University, Taipei, Taiwan, ROC

^c^Department of Biotechnology and Pharmaceutical Technology, Yuanpei University of Medical Technology, Hsinchu, Taiwan, ROC

^d^Graduate Institute of Medical Sciences, College of Medical Science and Technology, Taipei Medical University, Taipei, Taiwan, ROC

^e^Department of Primary Care Medicine, Taipei Medical University Hospital, Taipei, Taiwan, ROC

^f^Department of General Medicine, School of Medicine, College of Medicine, Taipei Medical University, Taipei, Taiwan, ROC

^g^TMU Research Center of Cancer Translational Medicine, Taipei Medical University, Taipei, Taiwan, ROC.

^h^Graduate Institute of Pharmacognosy, Taipei Medical University, Taipei, Taiwan, ROC

^#^ The first two authors contributed equally to this work.

Corresponding authors: Ming-Thau Sheu and Kuo-Hsiang Chuang

Address: 250 Wu-Hsing Street, Taipei 11031, Taiwan

E-mail: [mingsheu@tmu.edu.tw](mailto:mingsheu@tmu.edu.tw) and khchuang@tmu.edu.tw

Telephone and Fax: 886-2-23771942

**Method:**

**Physical Characterization of the DTX-Loaded** **L*_sb_*MDDs and BsAbs-L*_sb_*MDDs**

The average particle size and size distribution of both DTX-loaded L*_sb_*MDDs (2K or 5K) formulations were measured with an N5 submicron particle size analyzer (Beckman Coulter, Brea, CA, USA). The zeta potentials (ZPs) were measured with a Zetasizer (Nano-ZS) (Malvern Instrument, Worcestershire, UK). Transmission electron microscopy (TEM) was used to observe the surface morphology of the L*_sb_*MDDs; the aqueous L*_sb_*MDDs was adsorbed onto a grid and stained for 30 s with 2% (w/v) phosphotungstic acid (PTA). The grid was air-dried and observed by TEM (Hitachi H-700, Tokyo, Japan). The binding activity of DIO-loaded HER2-L*_sb_*MDDs after different storage times in PBS or FBS was determined by cellular uptake. The amount of encapsulated drug was detected with an HPLC method as previously validated (Sheu *et al.*, 2016). The HPLC conditions were as follows: an Inertsil 6 ODS-3 column (150 × 4.6 mm, 6 µm, Inertsil, GL Sciences, Tokyo, Japan); Jasco series equipment (pump PU-980, UV-975, Jasco, Tokyo, Japan); the composition of the mobile phase was acetonitrile/water (6/4, v/v); the flow rate was 1 mL/min; and the detection wavelength was 227 nm. The encapsulation efficiency (EE) and drug loading (DL) were, respectively, calculated as follows:

EE (%) = W_M_ / W_I_ × 100% and (1)

DL (%) = W_M_ / (W_P_ + W_M_) × 100%; (2)

where W_M_ is the amount of drug in the L*_sb_*MDDs, W_I_ is the amount of the initial feeding drug, and W_P_ is the amount of total polymers.

**Plasma analysis for DTX by UPLC/MS/MS**

The procedure for DTX extraction from plasma was as follows. The internal standard of 500 ng/mL paclitaxel in 20 μL was added to 200 μL plasma and 600 μL of acetonitrile was used for deproteinization with vigorous mixing for 5 min. After centrifugation at 10000 rpm for 10 min, the clear solution was collected, and the supernatant solution was transferred to PhreeTM (Phenomenex^®^) to eliminate potential interference in the plasma. The composition of the mobile phase was 0.1% formic acid and acetonitrile containing 0.1% formic acid at a flow rate 0.3 mL/min according to a method adapted from Sheu *et al.,*(Sheu *et al.*, 2016)*.* Electrospray ionization was performed in the positive ion model. The capillary voltage was 3.9 kV, the cone voltage was 65 V, the desolvation temperature was 500°C, the desolvation gas flow was 600 L/h, and the collision gas flow was 25 L/h. The PK parameters, including the plasma drug concentration-time profile from the area under the curve (AUC) at 0 to infinity (AUC_0-inf_), maximum plasma concentration (C_max_), half-life (T_1/2_), volume distribution (V), and clearance (Cl), were estimated using a non-compartmental method provided by WinNonlin 6.3 software (Pharsight^®^, Princeton, NJ, USA)

**The HER2 binding activity of HER2-L*_sb_*MDDs in the PK studies**

The experimental protocol to assess the HER2 binding activity of HER2-L*_sb_*MDDs is illustrated in Scheme 3 (supplemental information). First, MCF7/HER2 cells were seeded in 96 well at a density of 2×10^5^ cells per well at 37°C for overnight. The different time points of plasma samples (1, 4, 10, 24, 48 and 72 h) were diluted to same concentration of DTX (5 ng/mL) and added to cells. After incubation for 1 h, each well was washed with DMEM/F12 to remove unbound nanocarriers. Then mouse anti-PEG_backbone_ antibody (clone 6.3) (from Steve Roffler, Institute of Biomedical Sciences, Academia Sinica, Taiwan) and secondary antibody of goat anti-mouse immunoglobulin G (IgG) Fc-horseradish peroxidase (HRP) was added for another 1 h to detect the bind activity. The plates were finally washed with DMEM/F12 and then 150 μL/well of ABTS substrate was added for 60 min. Color development was measured at 405 nm (Bio-Tek, Winooski, VT, USA).


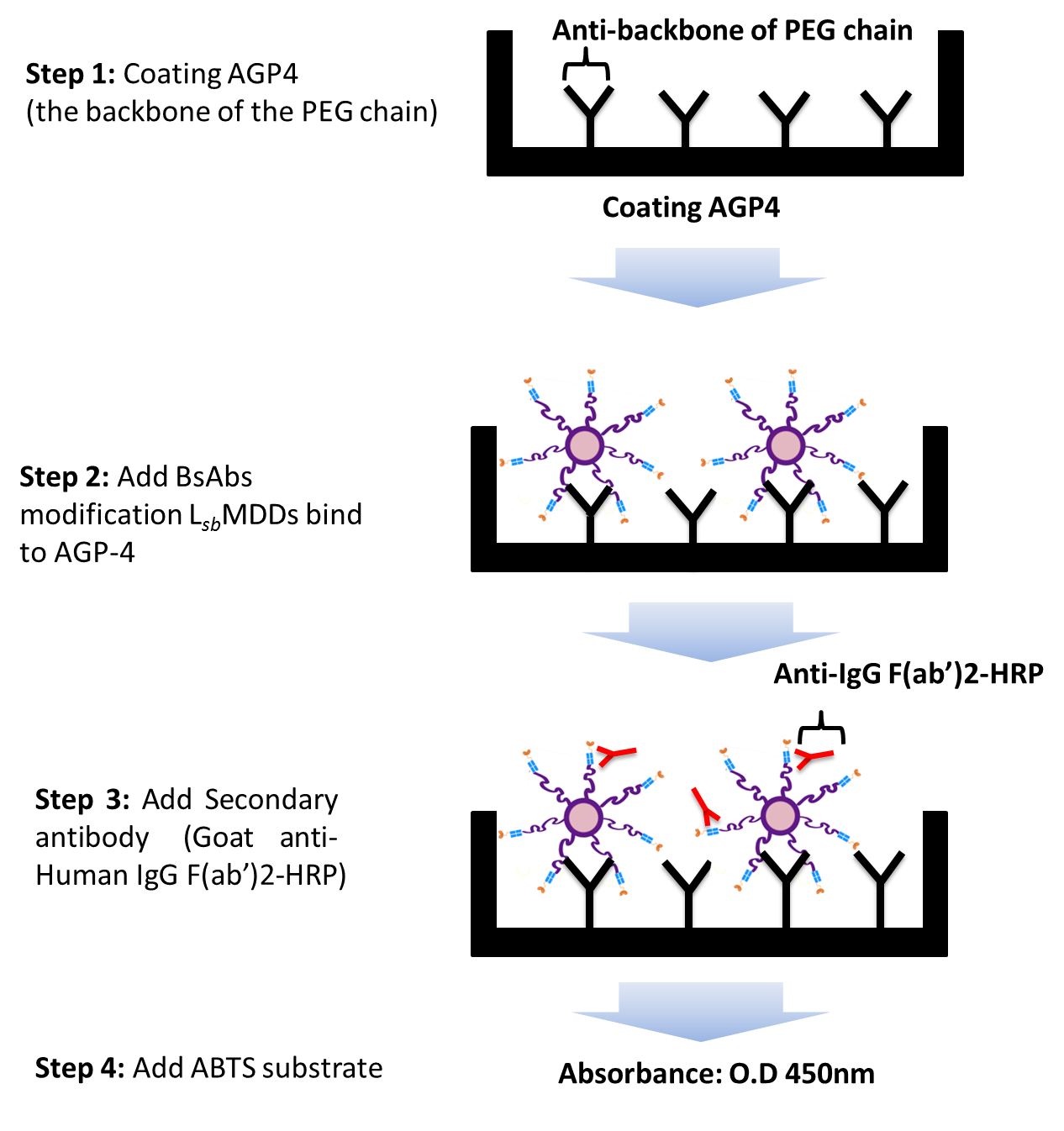


**Scheme 1**: Experimental protocol for detection of BsAbs (anti-mPEG/anti-HER2) on the L*_sb_*MDDs (2K or 5K) by a sandwich ELISA method


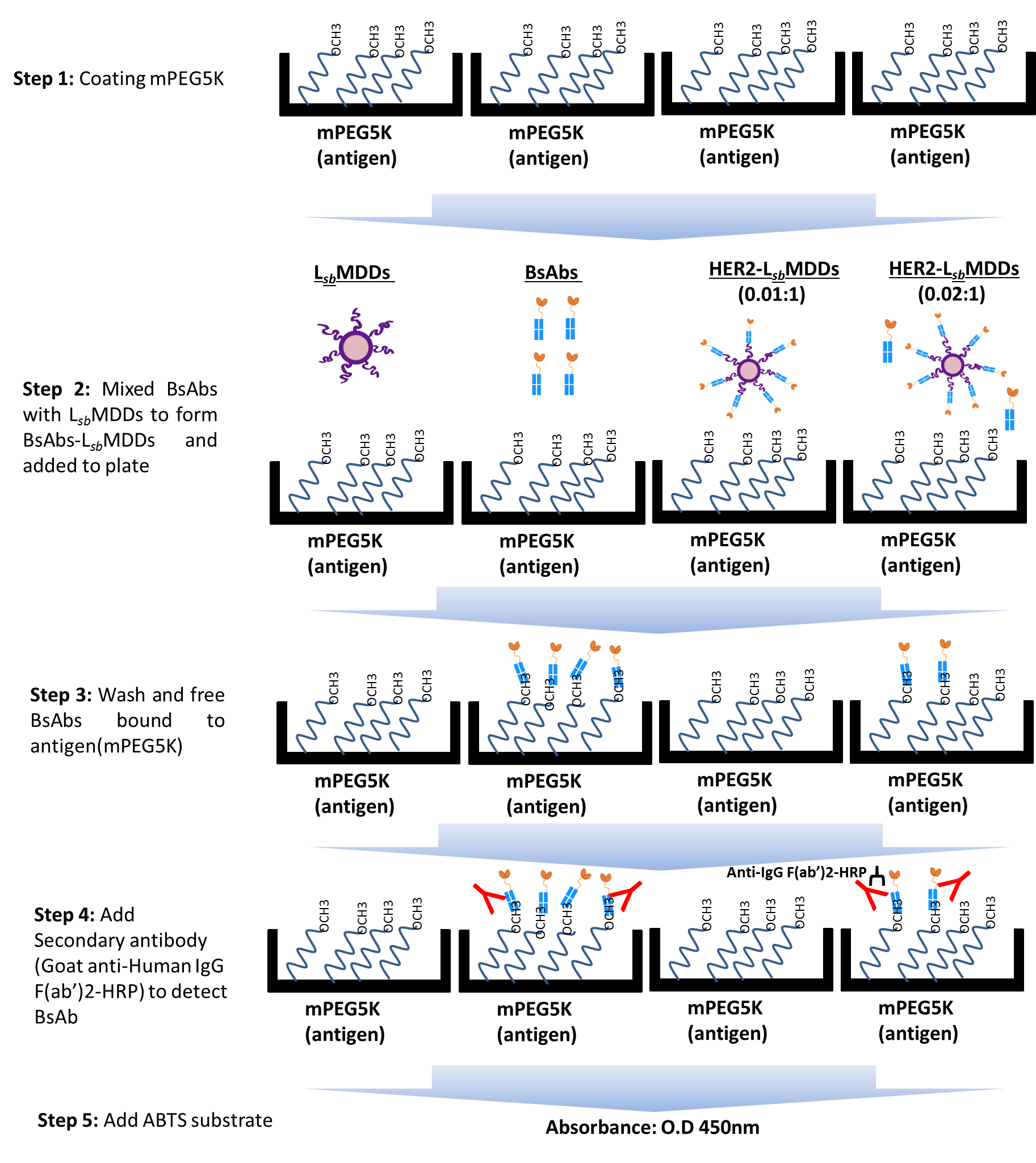


**Scheme 2**: Experimental protocol to optimize the molar ratio of BsAbs(anti-mPEG/anti-HER2) to mPEG5K on the L*_sb_*MDDs by an ELISA method.


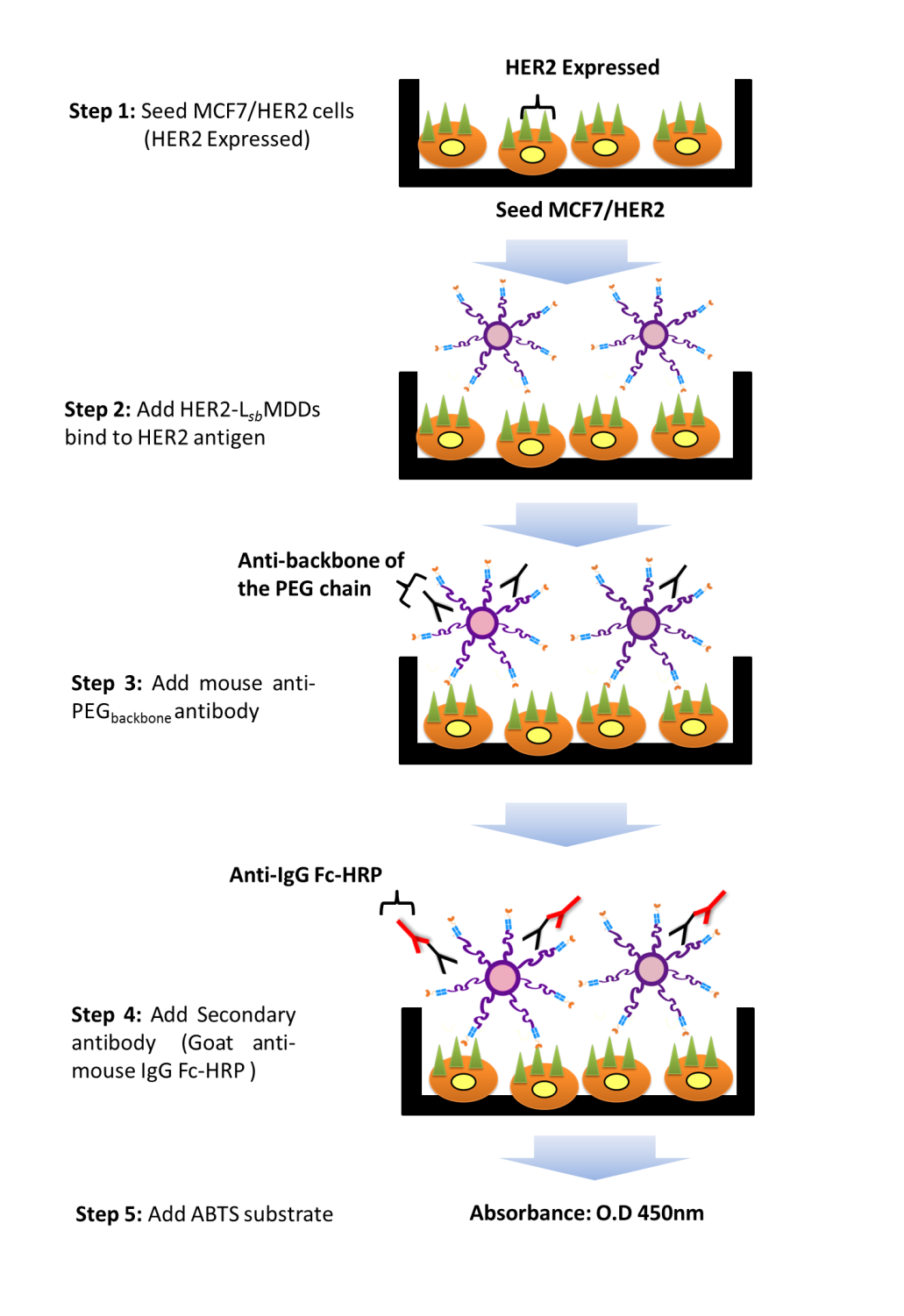


**Scheme 3**: Experimental protocol to determine binding activity of HER2-L*_sb_*MDDs in the plasma by a cell-based ELISA method.

| **Table S1**: Pharmacokinetic parameters of docetaxel (DTX) after intravenous administration to SD rats of Tynen^®^, L*_sb_*MDDs, DNS-L*_sb_*MDDs, and HER2-L*_sb_*MDDs | | | | | |
| --- | --- | --- | --- | --- | --- |
| Formulations | C0  (ng/mL) | T1/2(hr) | AUC_0-inf_  (hr*ng/mL) | V(L/Kg) | Cl(L/hr) |
| Tynen^®^ | 5196±1015 | 48.9±21.3 | 2583±147 | 216.5±87.7 | 3.1±0.17 |
| L*_sb_*MDDs | 15085±5497 | 24.1±9.5 | 2276±473 | 120.4±25.5 | 3.63±0.76 |
| DNS- L*_sb_*MDDs | 12949±6113 | 24.7±6.1 | 1928±466 | 101.4±80.6 | 5.09±1.59 |
| HER2- L*_sb_*MDDs | 9457±4454 | 25.3±10.4 | 2093±451 | 145.1±73.6 | 3.93±0.75 |


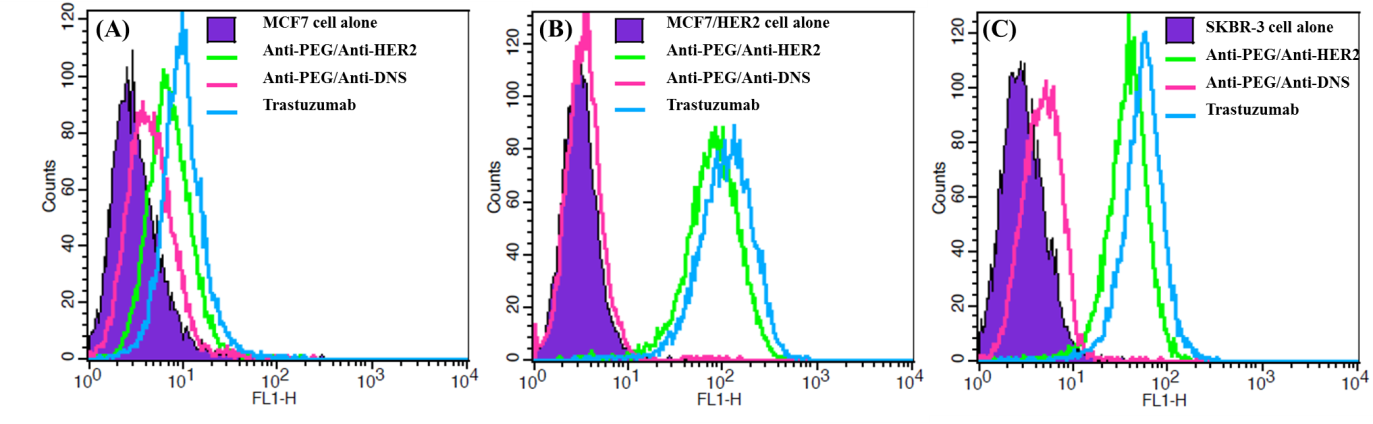


**Figure. S1.** The expression level of tumor markers and function of bispecific antibodies (anti-mPEG/anti-HER2 and anti-mPEG/anti-DNS) were examined on (A) MCF-7 (B) MCF-7/HER2 and (C) SKBR-3 breast cancer cells.


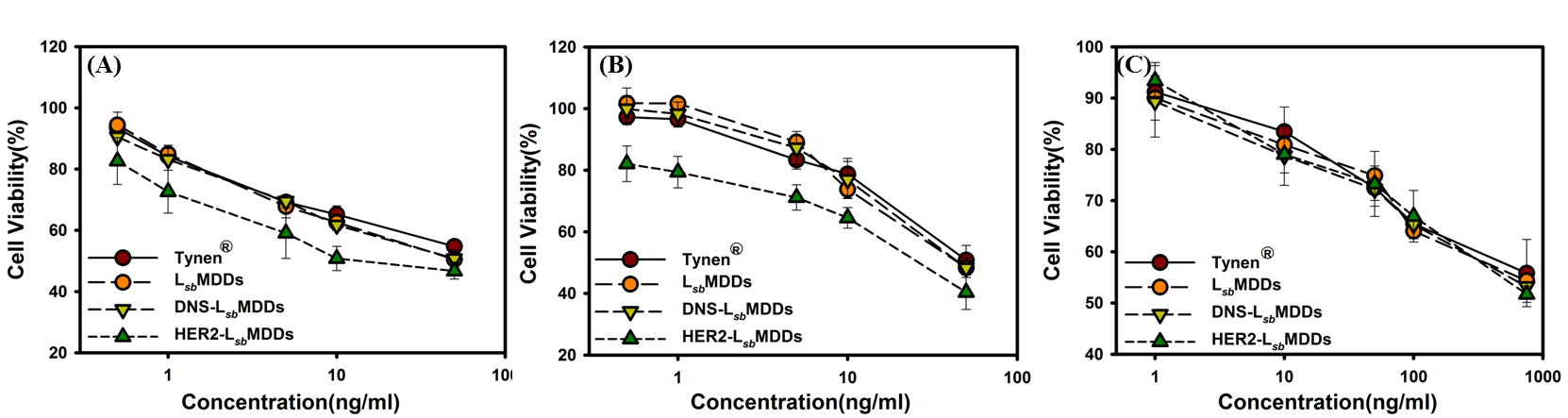


**Figure. S2.** Cell viabilities of Tynen^®^, the L*_sb_*MDDs, DNS-L*_sb_*MDDs, and HER2-L*_sb_*MDDs in the (A) MCF-7/HER2, (B) SKBR-3, and (C) MCF-7 cell lines.


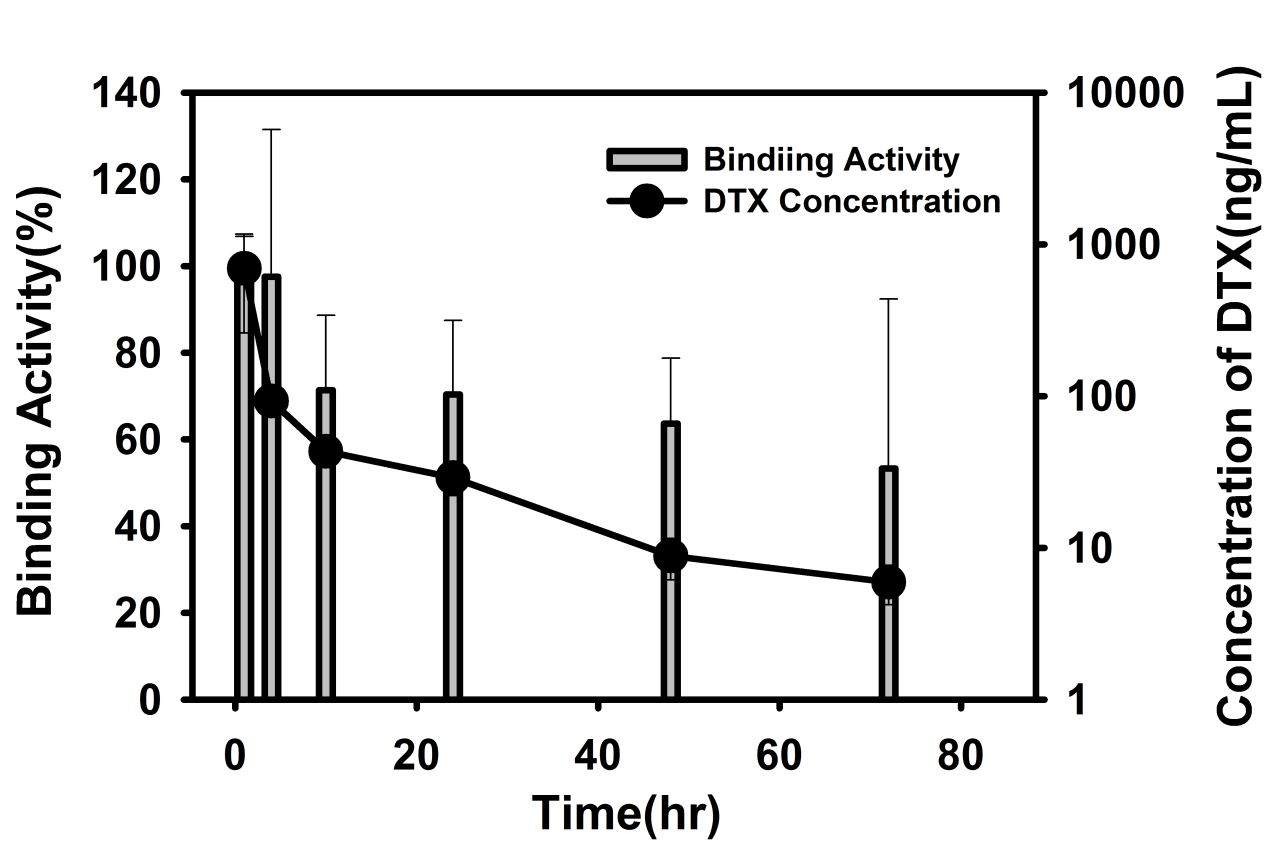


**Figure. S3.** The HER2 binding activity of HER2-L*_sb_*MDDs and plasma concentration-time curves of docetaxel after intravenous administration of HER2-L*_sb_*MDDs at a dose of 10 mg/kg to rats (*n*=3)**.** Binding activity is presented as the percentage compare to the 1hr.

**Reference:**

Sheu, M.T., Jhan, H.J., Su, C.Y., Chen, L.C., Chang, C.E., Liu, D.Z. & Ho, H.O., 2016. Codelivery of doxorubicin-containing thermosensitive hydrogels incorporated with docetaxel-loaded mixed micelles enhances local cancer therapy. *Colloids Surf B Biointerfaces,* 143**,** 260-270.
